# Application of modified alveolar ridge augmentation technique for horizontal bone augmentation in posterior mandibular region: Report of 3 cases

**DOI:** 10.1002/ccr3.2548

**Published:** 2019-11-22

**Authors:** Shunli Chu, Hongwei Xu, Xianjing Li, Tianqi Guo, Zhu Ting, Yanmin Zhou

**Affiliations:** ^1^ Department of Dental Implantology School and Hospital of Stomatology Jilin University Changchun City China

**Keywords:** bone splitting, compromised bone quantity, dental implants, horizontal bone augmentation, ultrasonic osteotomy technique

## Abstract

Three cases with severe horizontal bone deficiency on mandibular posterior region were committed by modified alveolar ridge augmentation. The therapeutic outcomes show that it is an effective methodology in cases with compromised horizontal bone.

## Graphical abstract

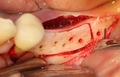

## 1 INTRODUCTION

Up until the present, dentition defects raised by endodontic and periodontal diseases are common concerns in clinical practices. Implant prosthodontics are shown with good masticatory efficiency, neglectable foreign body sensation, and do not damage the adjacent teeth as the traditional porcelain/metal bridge prosthodontics. However, the alveolar bone resorption often occurs associated with the defect of dentition, this is ascribed to the fact that functional loading by intact dentition is the prerequisite for maintaining the bone structures, without effective stimulation by masticatory forces, alveolar bone is prone to atrophy resorption.^1^ The resorption of alveolar ridge mainly occurs adjacent to three regions: periodontal membrane, periosteum of buccal‐lingual bone plate, and within the marrow cavity at cancellous bones.^2^ The buccal side of bone plate at mandibular posterior region is thinner than in lingual side, and the mandibular bone resorption always result in the insufficient bone quantity at horizontal direction. As an effective method for horizontal bone augmentation, the bone‐splitting technique does not need autologous bone grafting; however, the surgical trauma of traditional bone splitting is still unneglectable and unexpected bone fractures are also shown to happen during the splitting surgery.^3^ To overcome these disadvantages, we designed a modified bone‐splitting technique, and applied them on 3 cases with insufficient bone width at mandibular posterior region, this paper will discuss these cases in detail and provide future perspectives for such modified technique.

## 2 CASE 1

**Figure 1:**
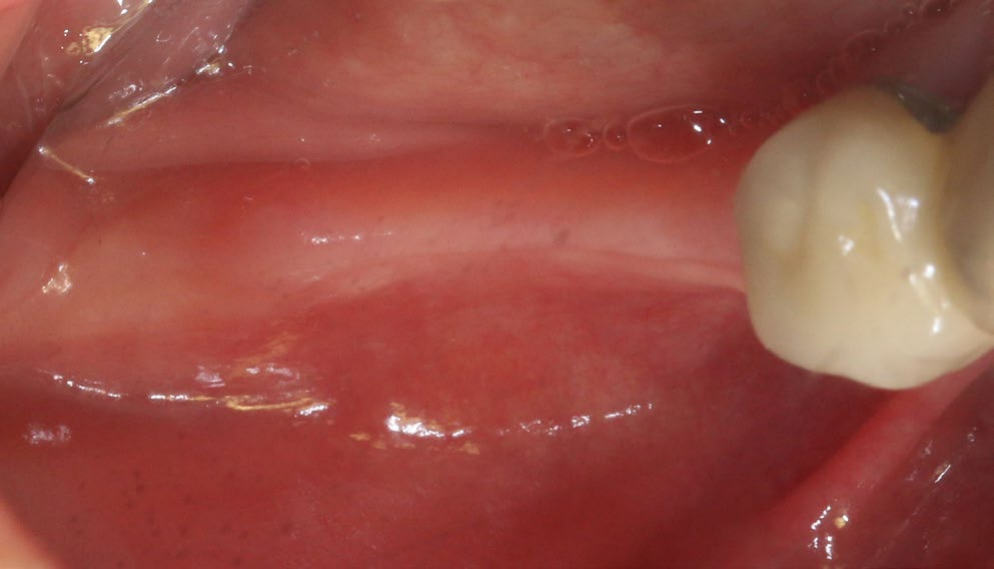
Teeth loss with horizontal bone insufficiency

**Figure 2:**
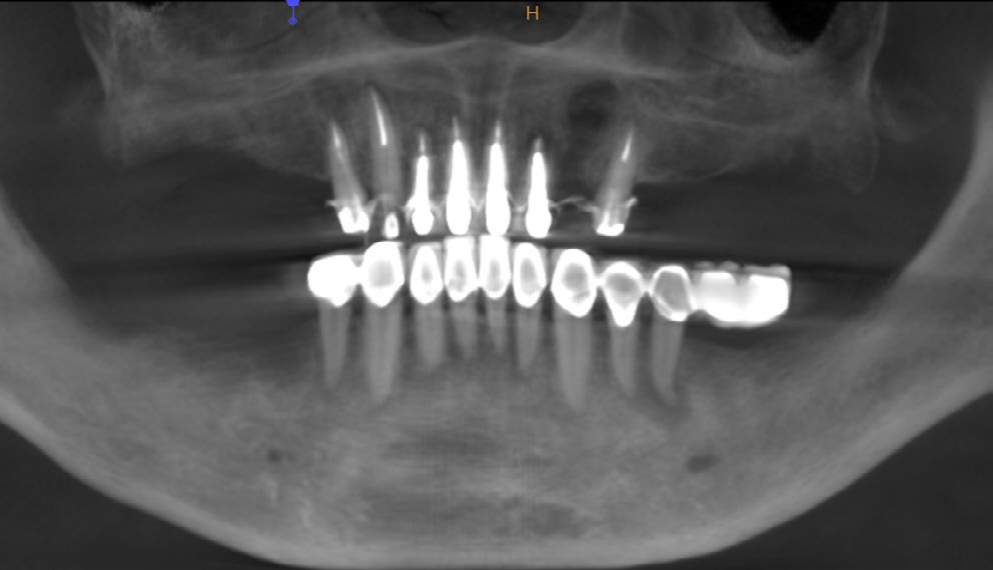
Panoramic view

**Figure 3:**
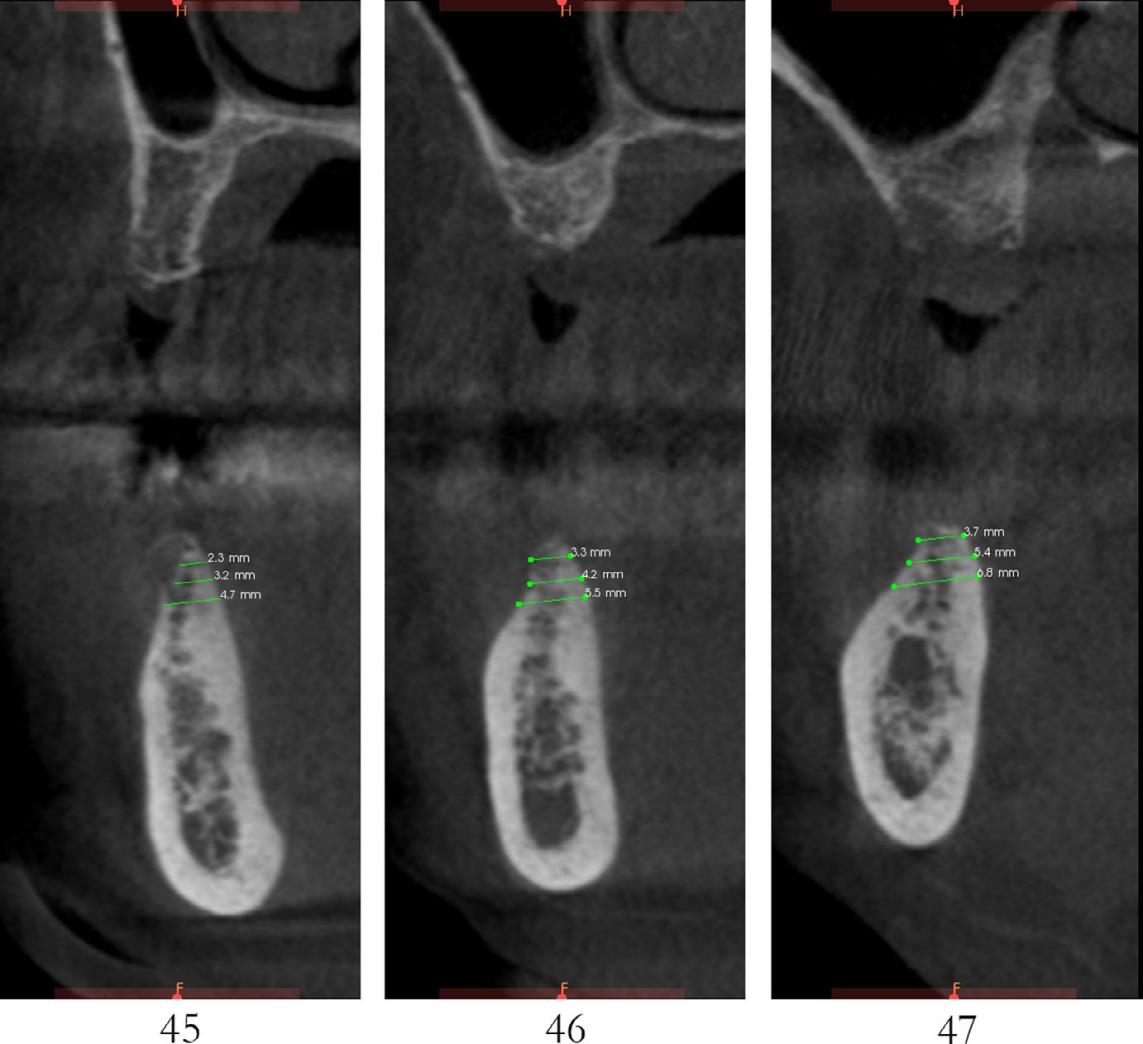
Residual bone crest (#45,#46,#47)

Case 1 was a 48‐year‐old female patient with no systemic diseases, the bilateral posterior tooth were loss (Figure 1), which seriously compromised the daily mastications. Clinical and radiology examination indicated that the bone quantity of maxillary and left mandibular regions is sufficient for implant placement; however, the bone width at right mandibular region is significantly limited, moreover, the residual alveolar crest shaped as a narrow blade. CBCT scanning revealed that 45, 46, and 47 were missing, the vertical height of alveolar bone was sufficient but the horizontal width of alveolar crest was merely 3‐4 mm (Figures 2 and 3).

### 2.1 Treatment plan

The modified bone‐splitting technique with simultaneous GBR was planned at the posterior region of right mandible, and delayed implant placement was designed at 6 months after GBR. Conventional implant prosthodontics were designed for other missing teeth.

### 2.2 Treatment procedures

**Figure 4:**
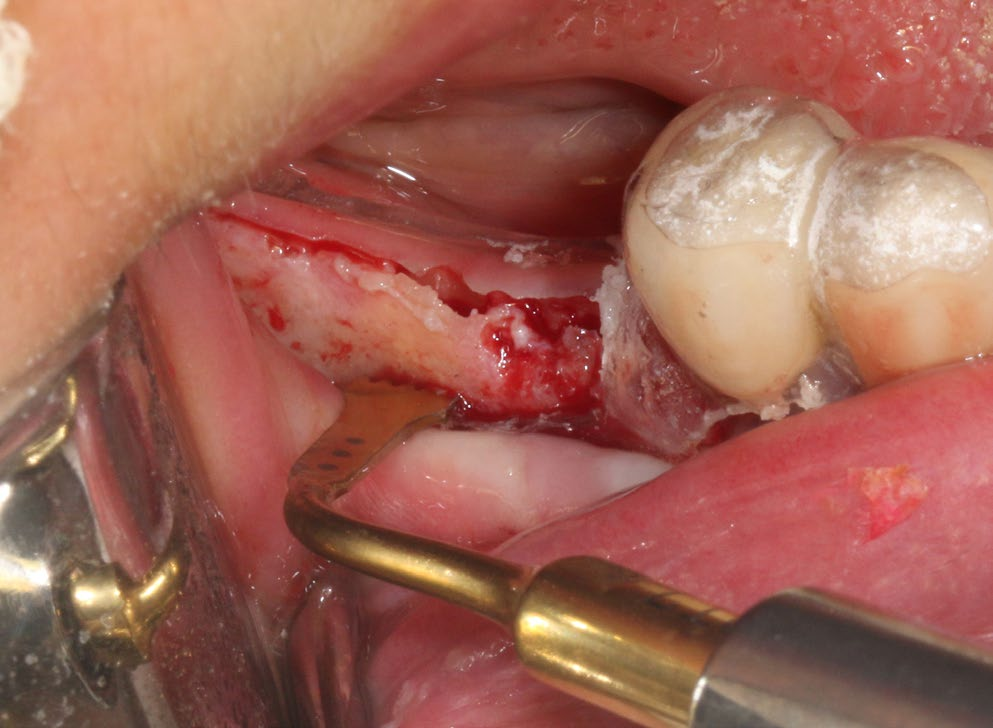
The linear cortical incision by PIEZOSURGERY

**Figure 5:**
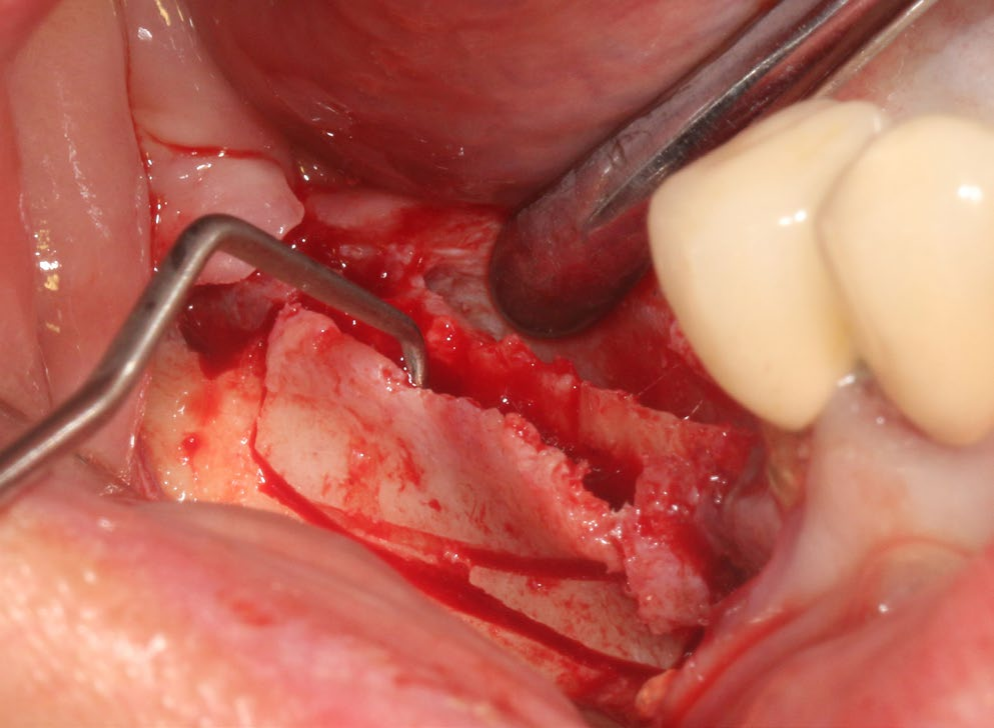
Bone dilatation

**Figure 6:**
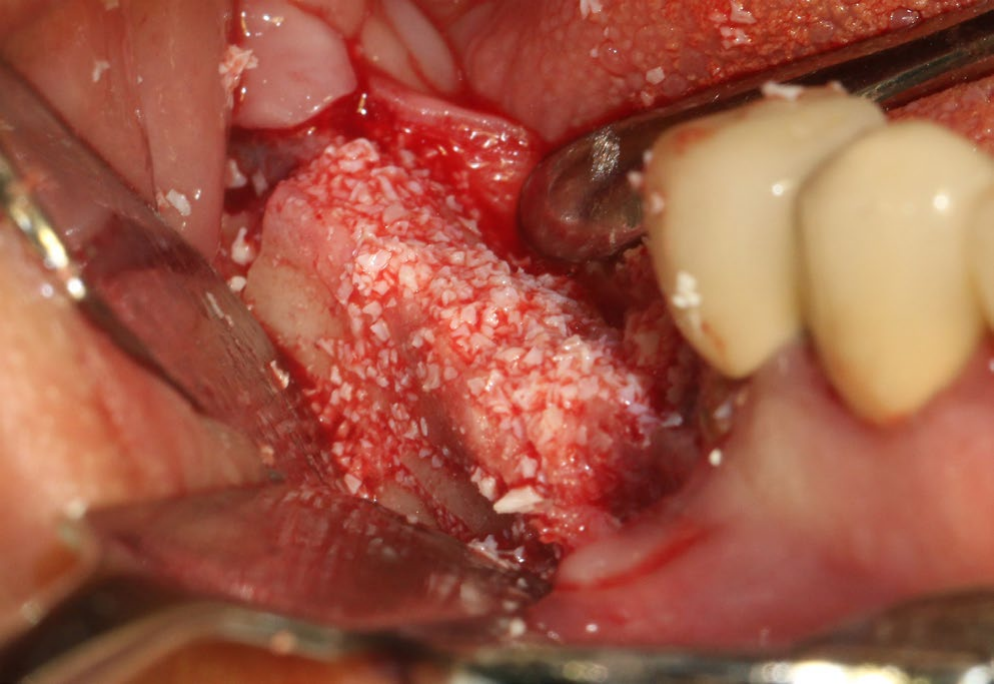
Sandwich bone substitute filling

**Figure 7:**
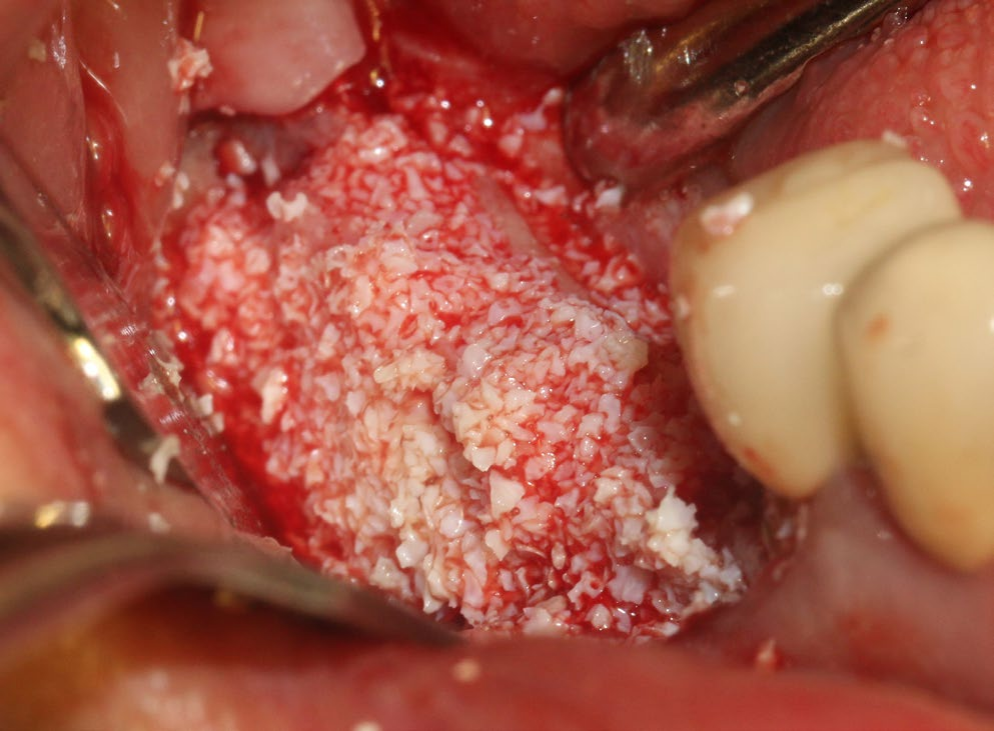
Bone substitute filling buccally

**Figure 8:**
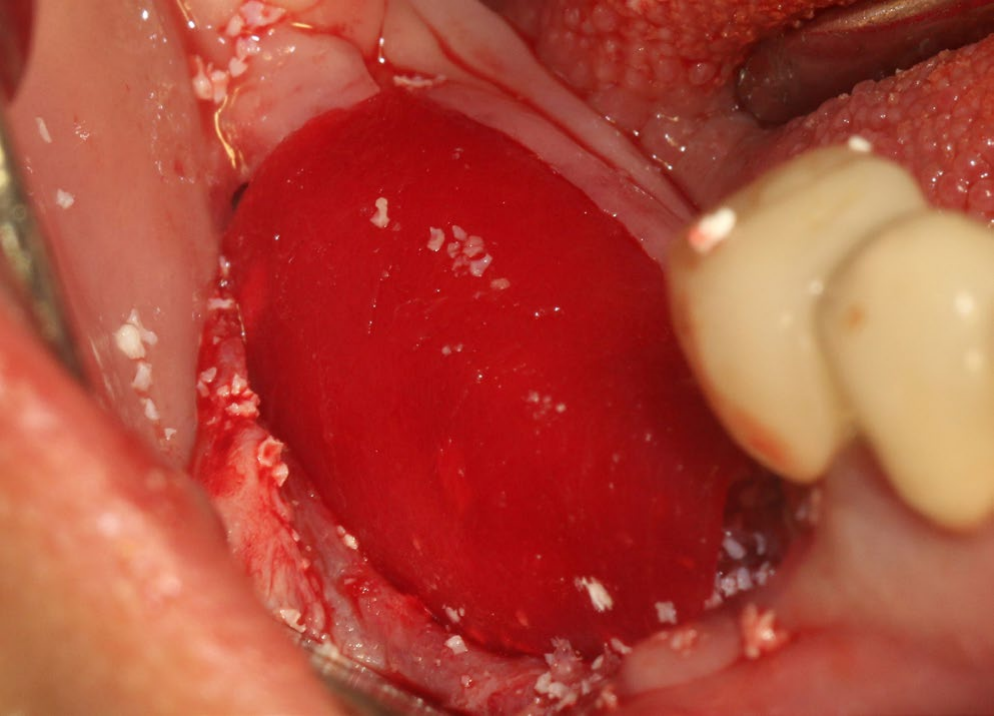
Covered by biofilm

**Figure 9:**
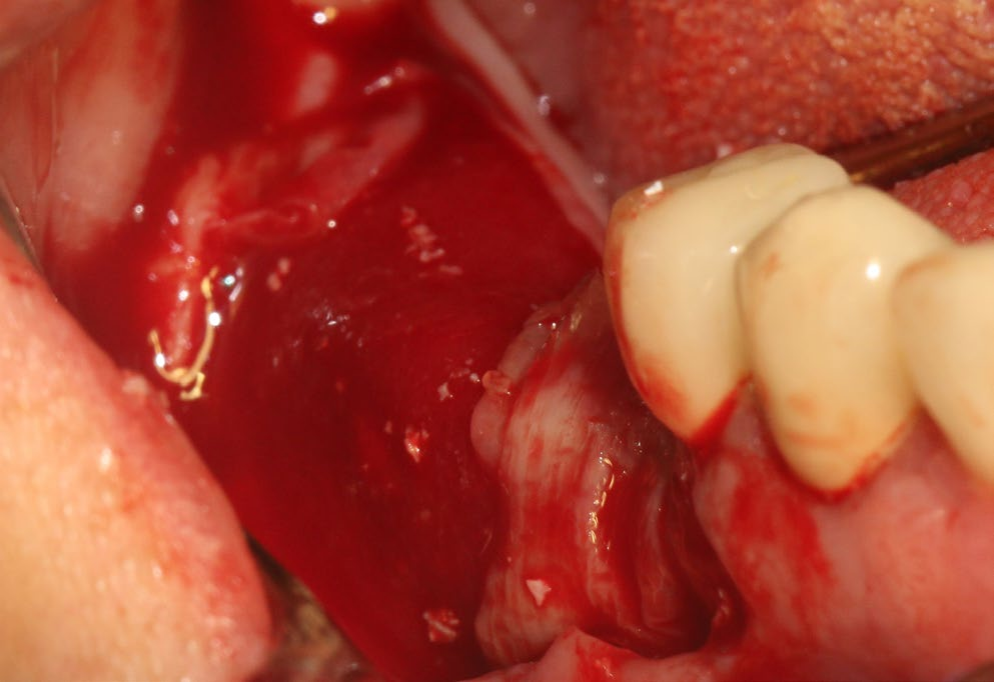
Covered by CGF membrane

**Figure 10:**
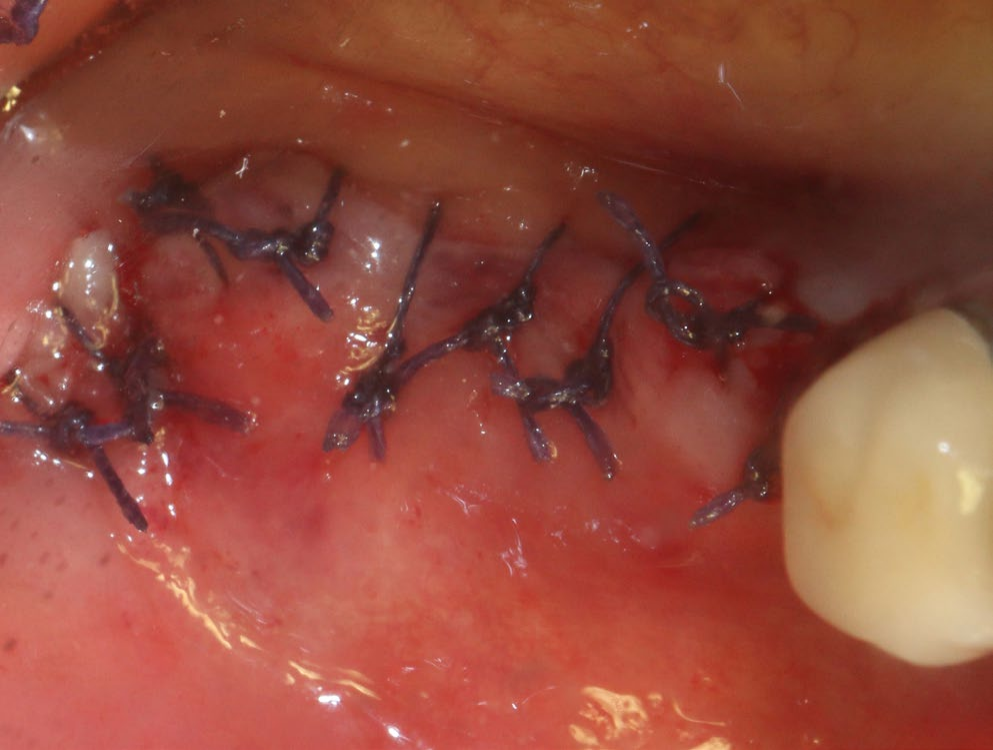
Suture the wound

After the disinfection and local anesthesia, the linear incision was proceeded along the “blade shaped” top of alveolar ridge by the piezosurgical blade (PIEZOSURGERY^®^ 3; Mectron S.p.A.) on the mesial‐distal direction. At the mesial and distal ends of top incision, 2 incisions were proceeded perpendicularly, and another A horizontal incision was cut 10 mm parallel to the top incision, all these 4 incisions penetrated through the cortical bone to reach the cancellous bone and bone marrow (Figure 4). Bleeding holes were prepared on the surface of the bone‐splitting region. A 4‐mm wide osteotome was inserted from the crestal incision and extended into the cancellous bone, the osteotome was gently pushed by bone chisel for approximately 8 mm, and linear bone expansion was processed at buccal‐lingual direction (Figure 5). Bio‐oss bone substitutions were added in the expanded area (Figures 6 and 7) and covered by a resorbable membrane (Haiao Heal‐ALL, ZH‐BIO) (Figure 8), a piece of PRF membrane (Figure 9) was attached on the top of the resorbable membrane to provide cytokines for bone/soft tissue regeneration. Finally, the surgical region was closed by simple interrupted suture(H‐563; Holycon) (Figure 10). Patients were instructed to use clindamycin and ornidazole for 7 days to prevent infections; the sutures were finally removed after 14 days.

**Figure 11:**
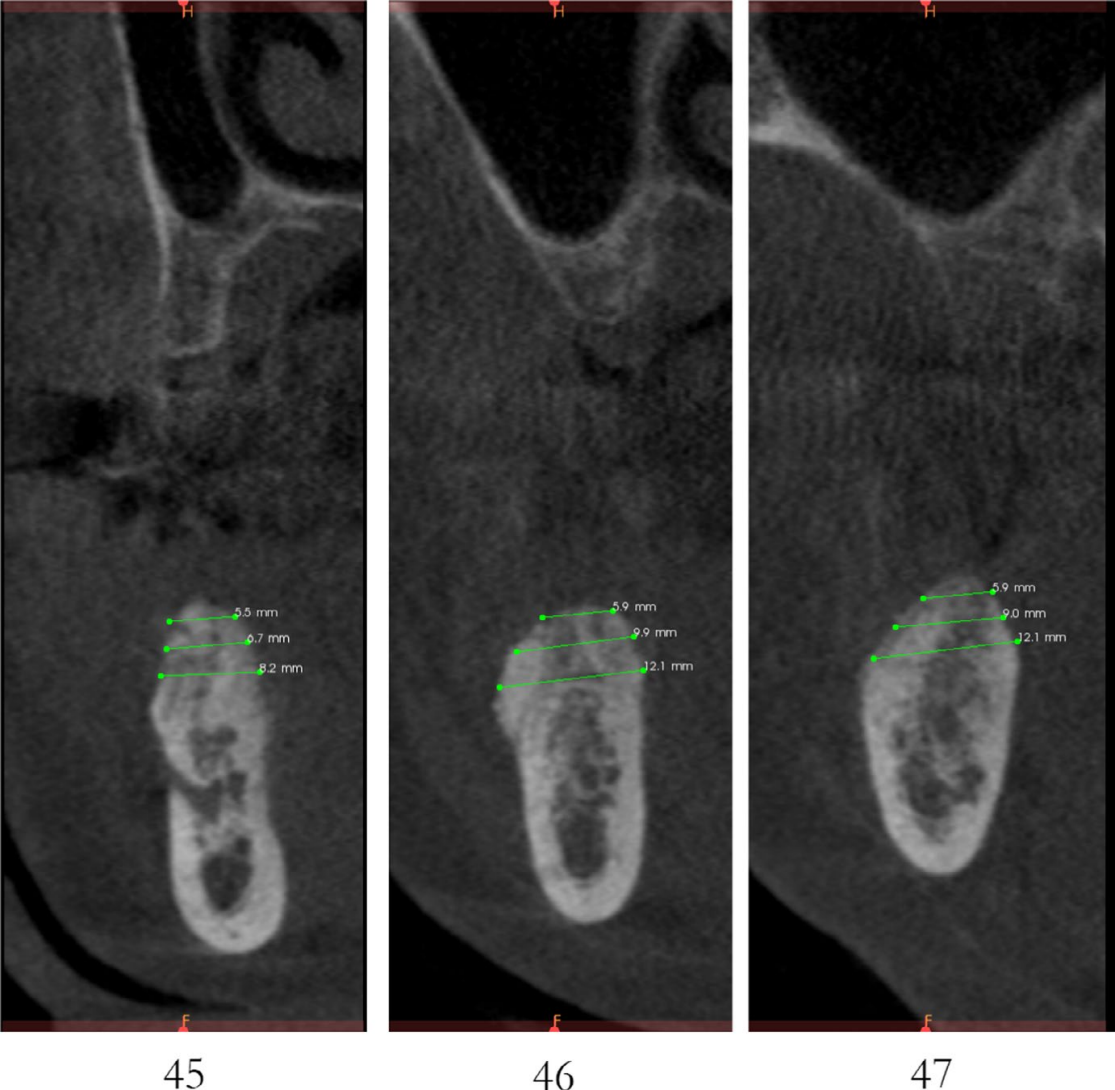
CBCT scanning 6 mo after the bone operation

**Figure 12:**
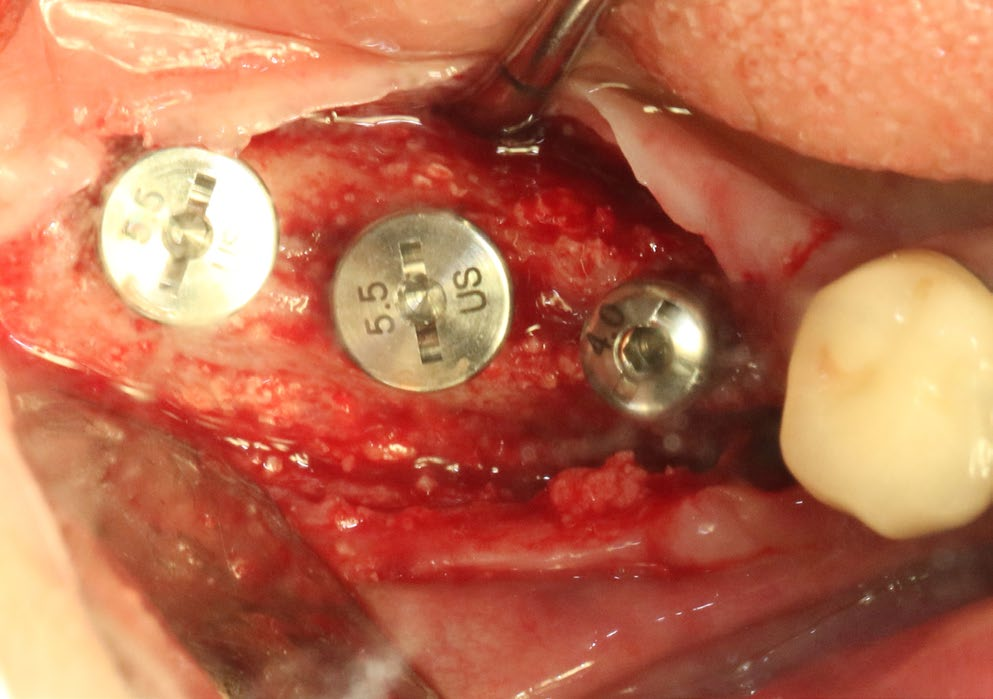
Three Dentium implants installed

**Figure 13:**
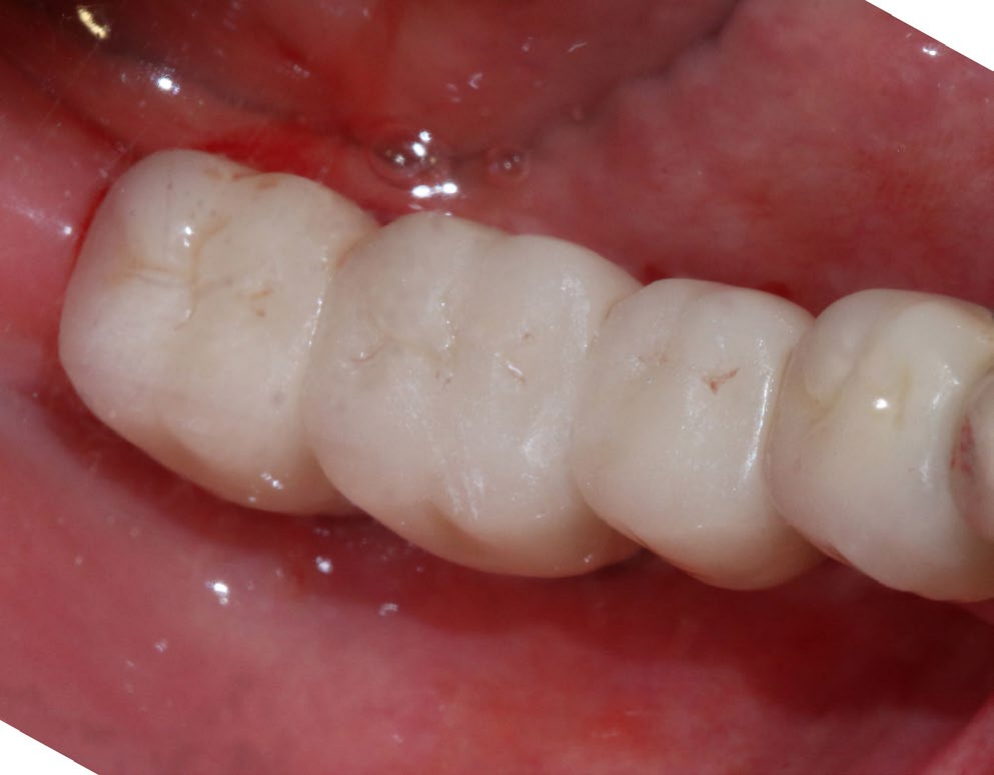
Final restoration

**Figure 14:**
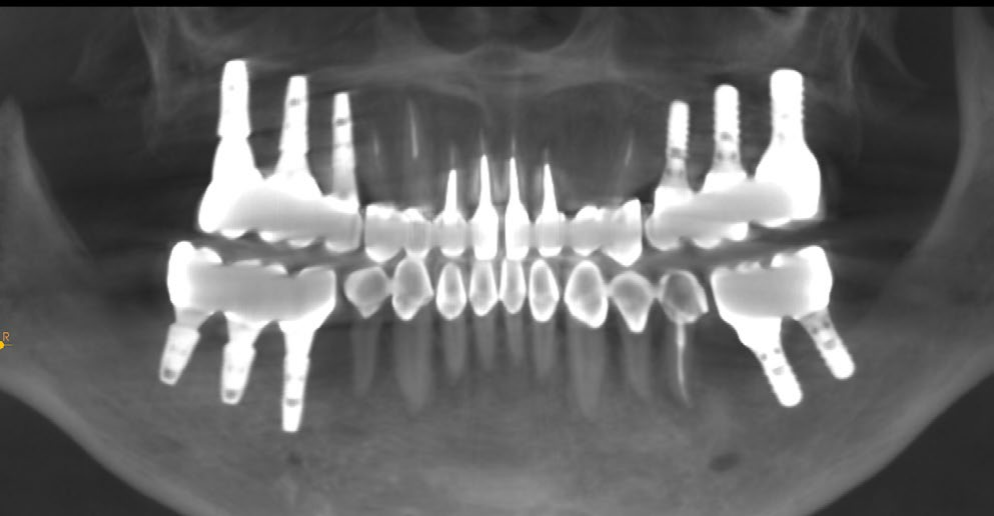
Panoramic 2 y after the operation

CBCT imaging at 6 months showed that an average augmentation of 2‐3 mm in alveolar ridge width was achieved at surgical site (Figure 11). With sufficient bond quantity, conventional dental implantation was performed under local anesthesia, and 3 implants were inserted (#45:3.6 mm(ф) × 10 mm(L), #46:4.5 mm(ф) × 8 mm(L), #47:4.5 mm(ф) × 8 mm(L); Dentium) (Figure 12). The final prosthodontics were finished at 3 months after implant placement (Figures 13 and 14).

### 2.3 Therapeutic effect

**Figure 15:**
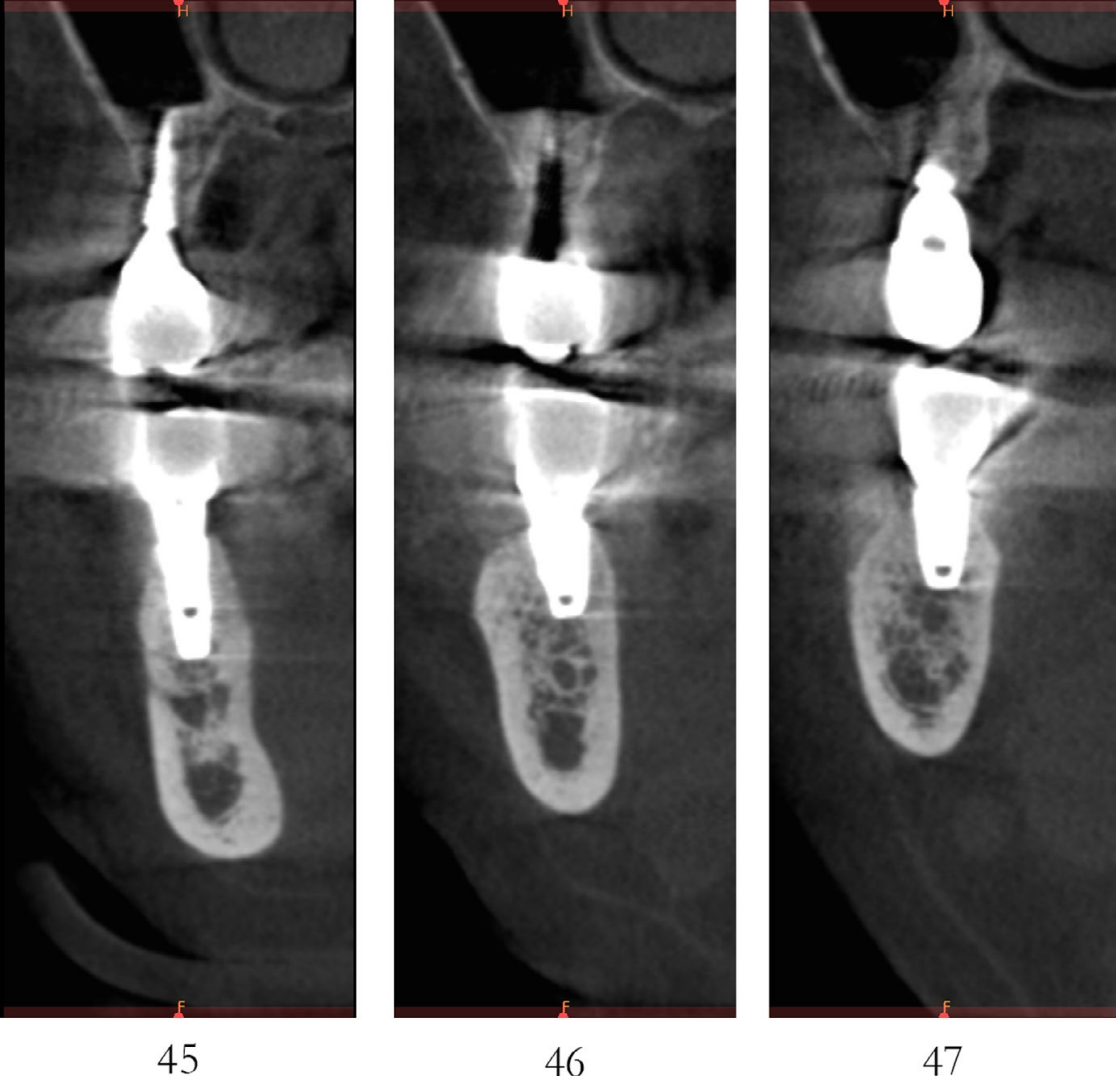
CBCT scanning 2 y after final restoration

**Figure 16:**
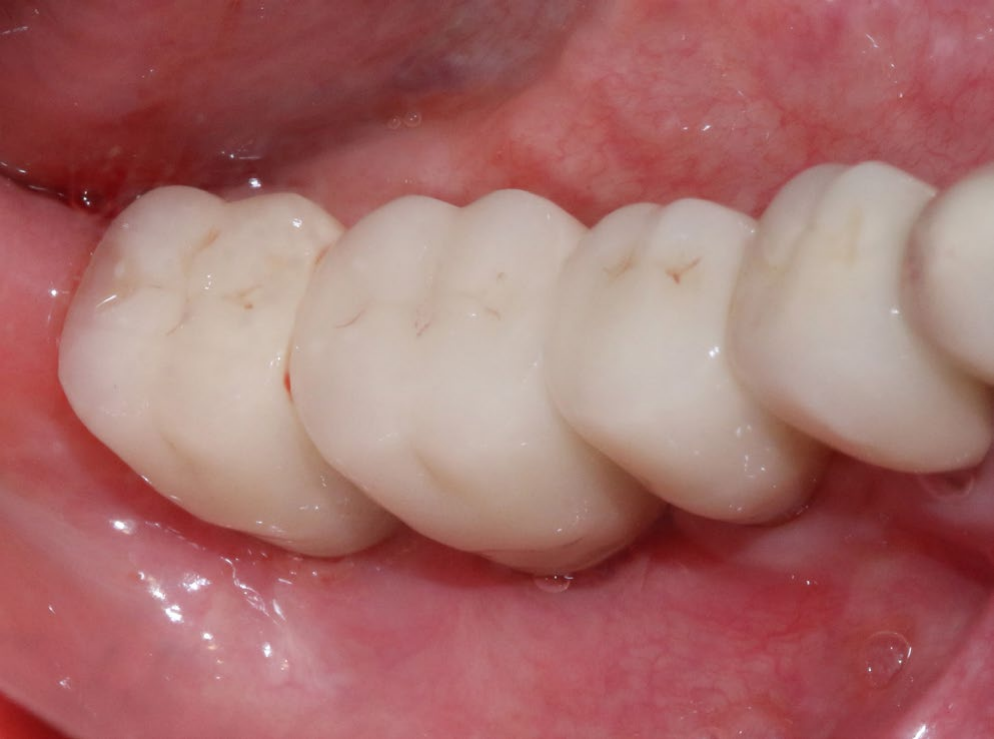
Return visit 2 y after final restoration

Significant bone width increment was achieved by the combination of modified piezosurgery technique and simultaneous GBR. The bone width was shown to be stable during and after 2 years of final restoration, and no significant bone resorption was revealed (Figures 15 and 16).

## 3 CASE 2

**Figure 17:**
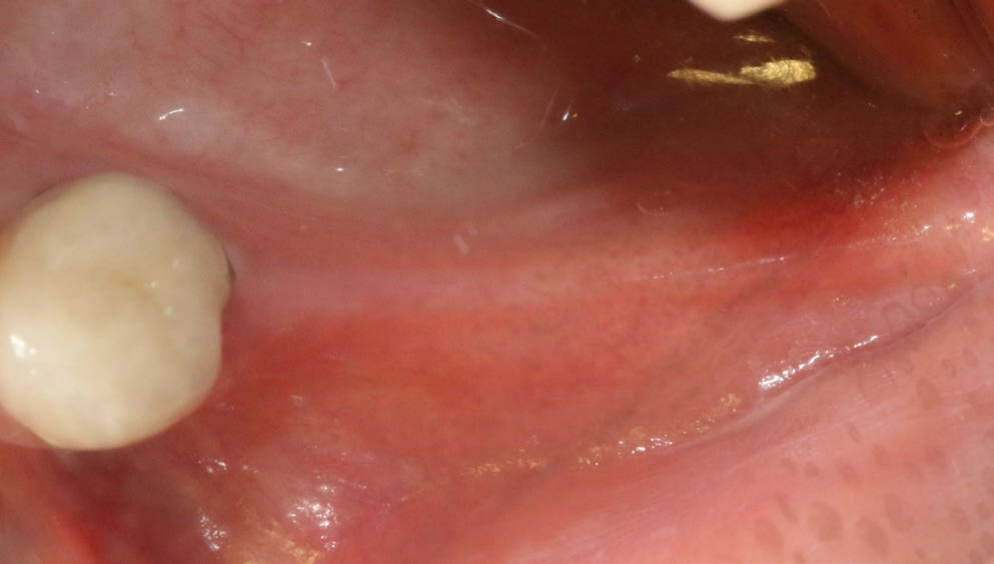
Teeth loss with horizontal bone insufficiency

**Figure 18:**
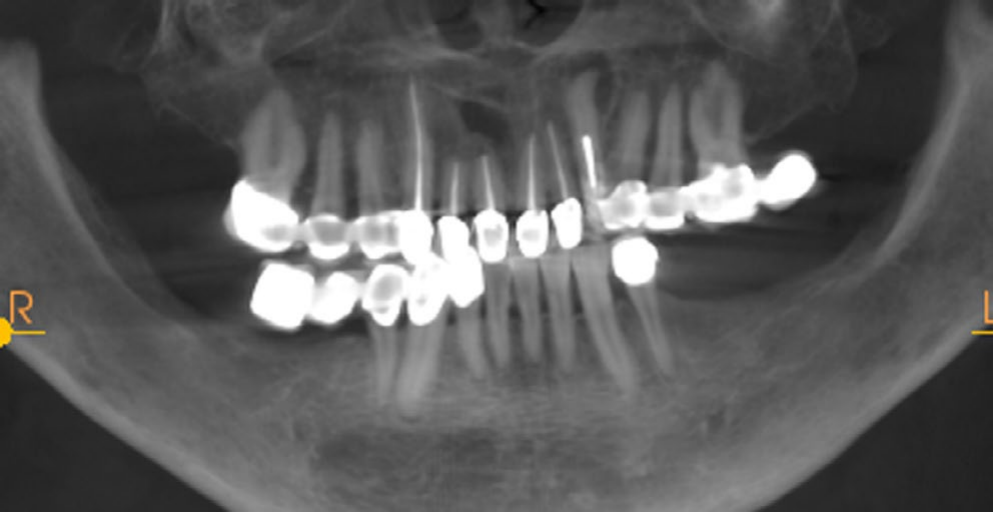
Panoramic view

**Figure 19:**
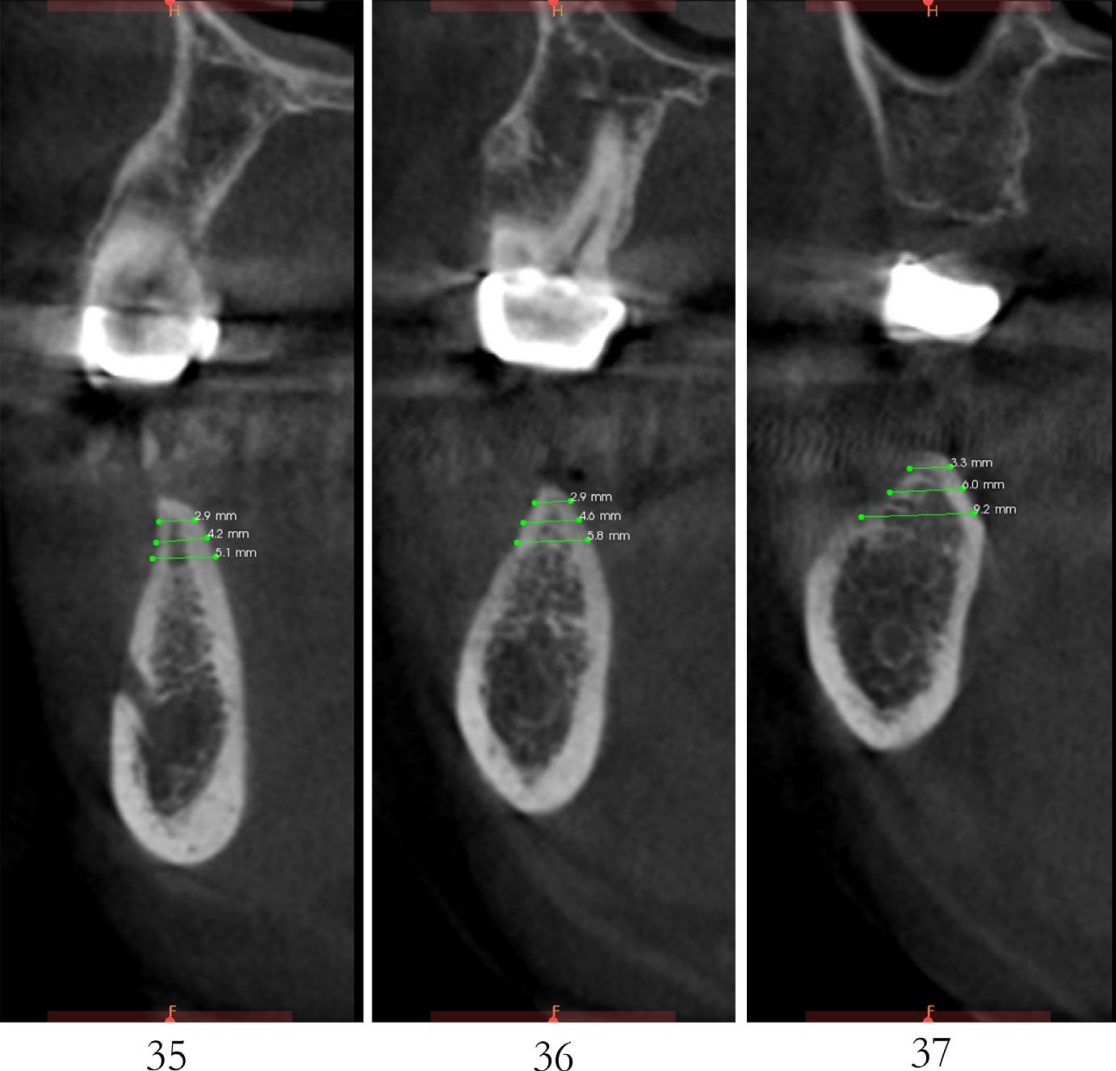
Residual bone crest (#35,#36,#37)

Case 2 was a 58‐year‐old woman without systemic disease. The 35, 36, and 37 were missing for the severe dental caries (Figure 17). CBCT showed the edentulous region of 35, 36, and 37 had a “blade shaped” residual alveolar ridge which is significantly insufficient for conventional implant placements, the width of residual alveolar crest was merely 3‐4 mm (Figures 18 and 19).

### 3.1 Treatment plan

The modified bone‐splitting technique with simultaneous GBR was planned at the posterior region of left mandible, and delayed implant placement was designed at 6 months after GBR.

### 3.2 Treatment procedures

**Figure 20:**
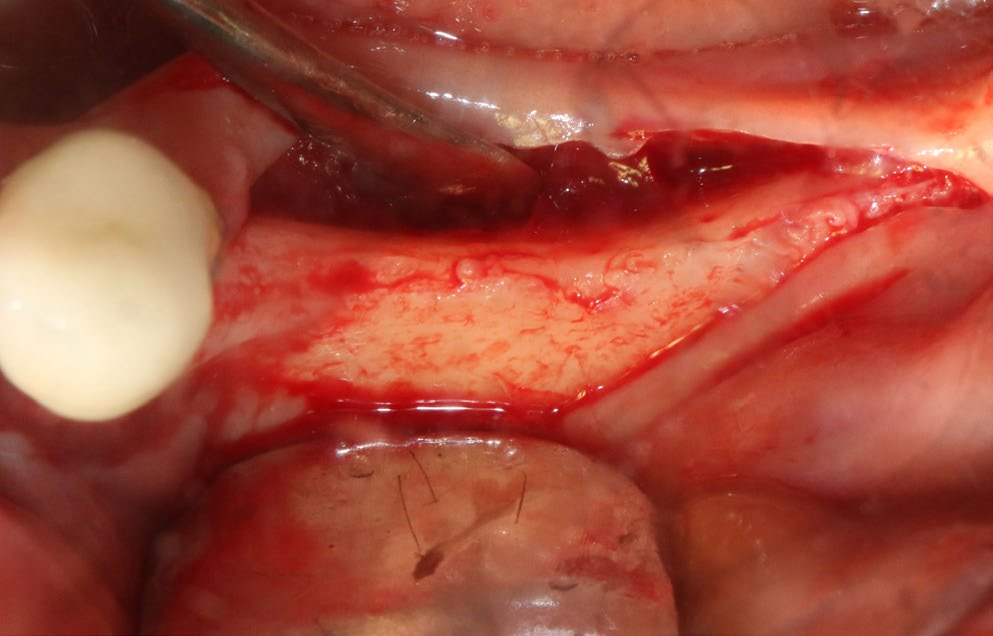
Thin bone crest

**Figure 21:**
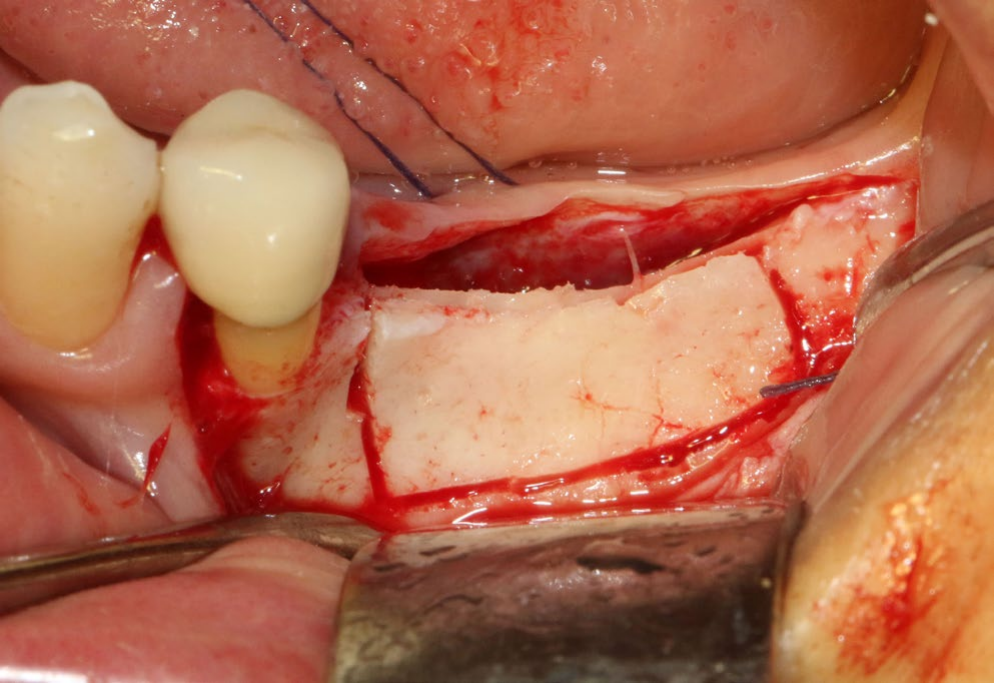
The linear cortical incision by PIEZOSURGERY

**Figure 22:**
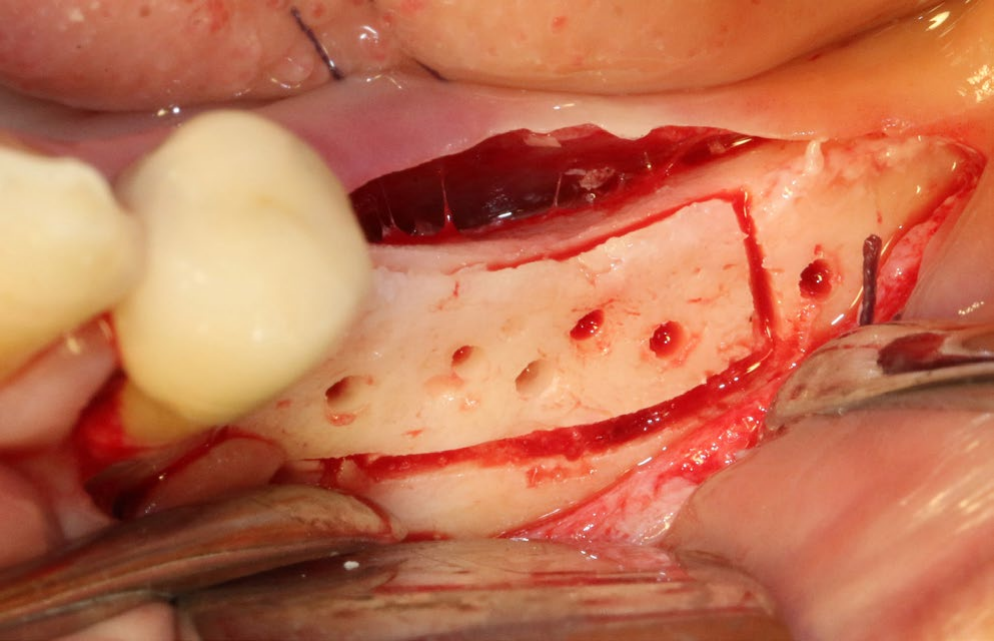
Bleeding holes

**Figure 23:**
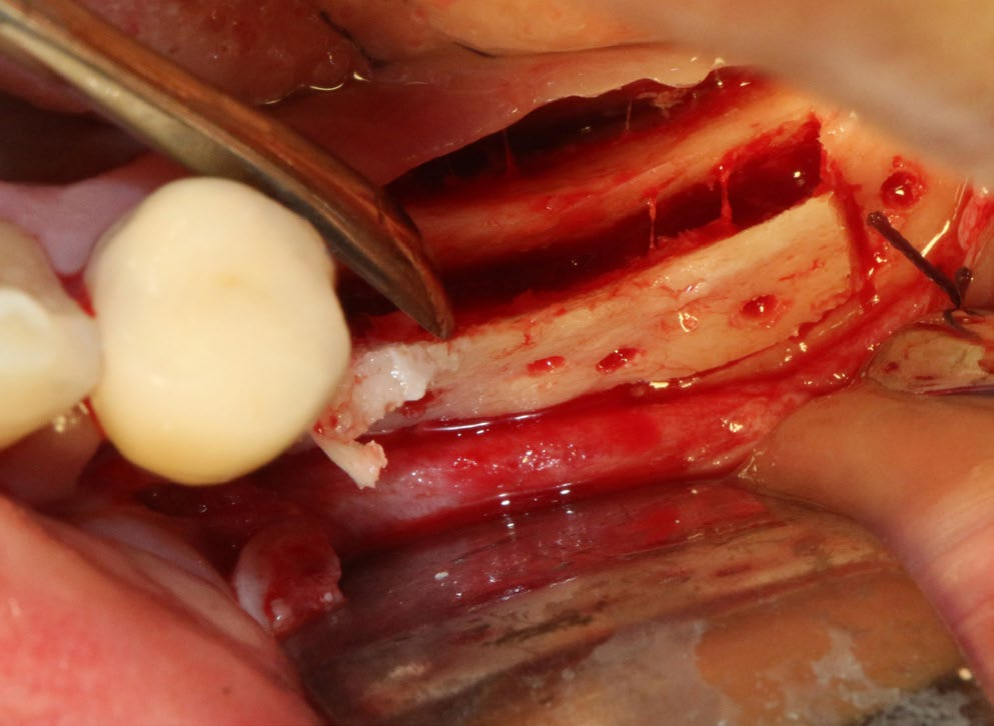
Bone dilatation

**Figure 24:**
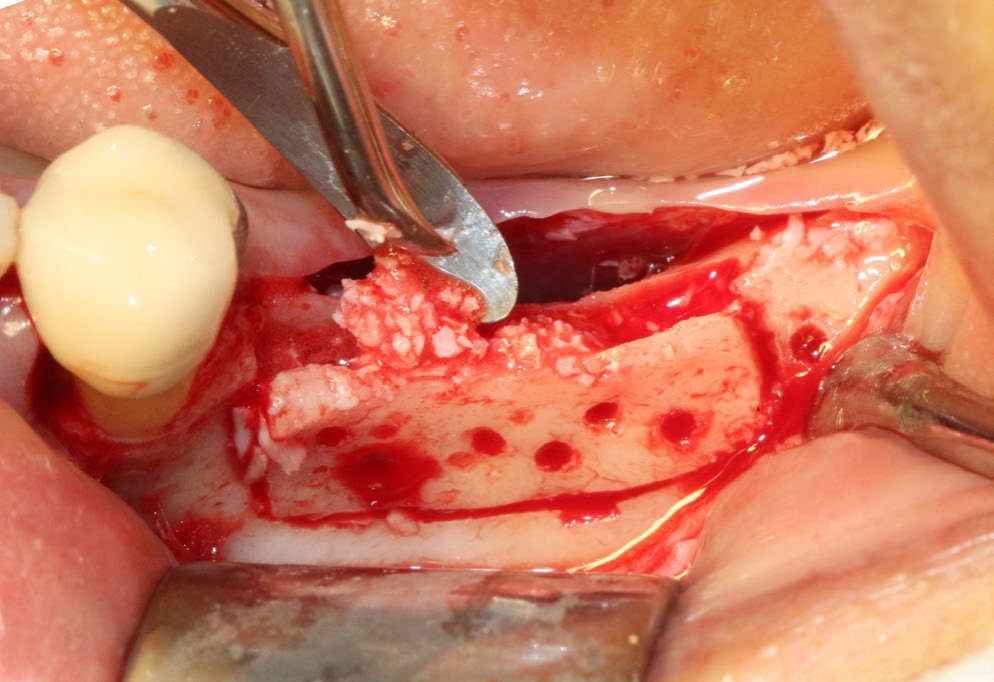
Sandwich bone substitute filling

**Figure 25:**
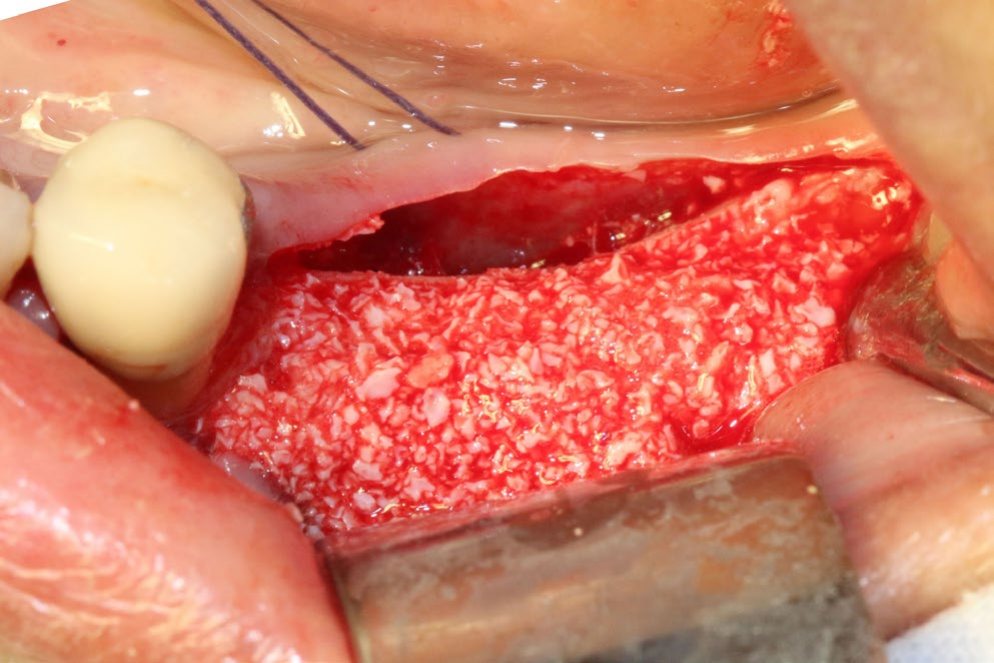
Bone substitute filling buccally

**Figure 26:**
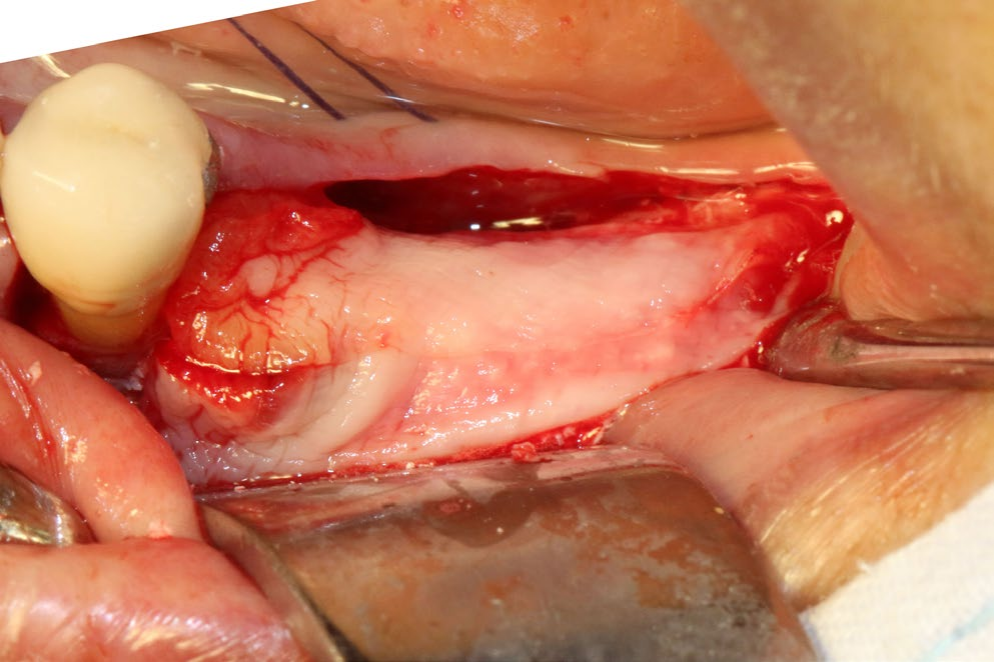
Covered by CGF membrane

**Figure 27:**
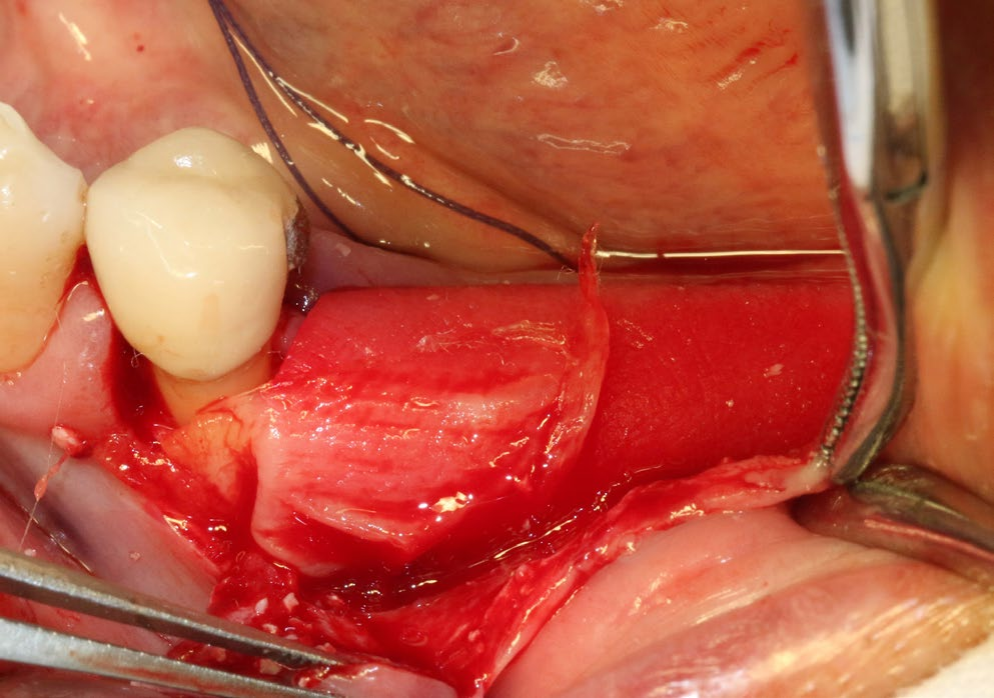
Covered by biofilm and CGF

After disinfection and local anesthesia, the same alveolar ridge augmentation was performed at 35‐37 sites as in case 1 (Figures 20, 21, 22, 23). After the bone splitting, Bio‐oss bone substitution was added into the bone‐splitting cavity (Figures 24 and 25), and covered by two pieces of PRF membrane and the resorbable membrane (Haiao Heal‐ALL, ZH‐BIO) (Figures 26 and 27). Surgical sites were closed with tight suture, and patient was instructed to take clindamycin and ornidazole for 7 days against infection.

**Figure 28:**
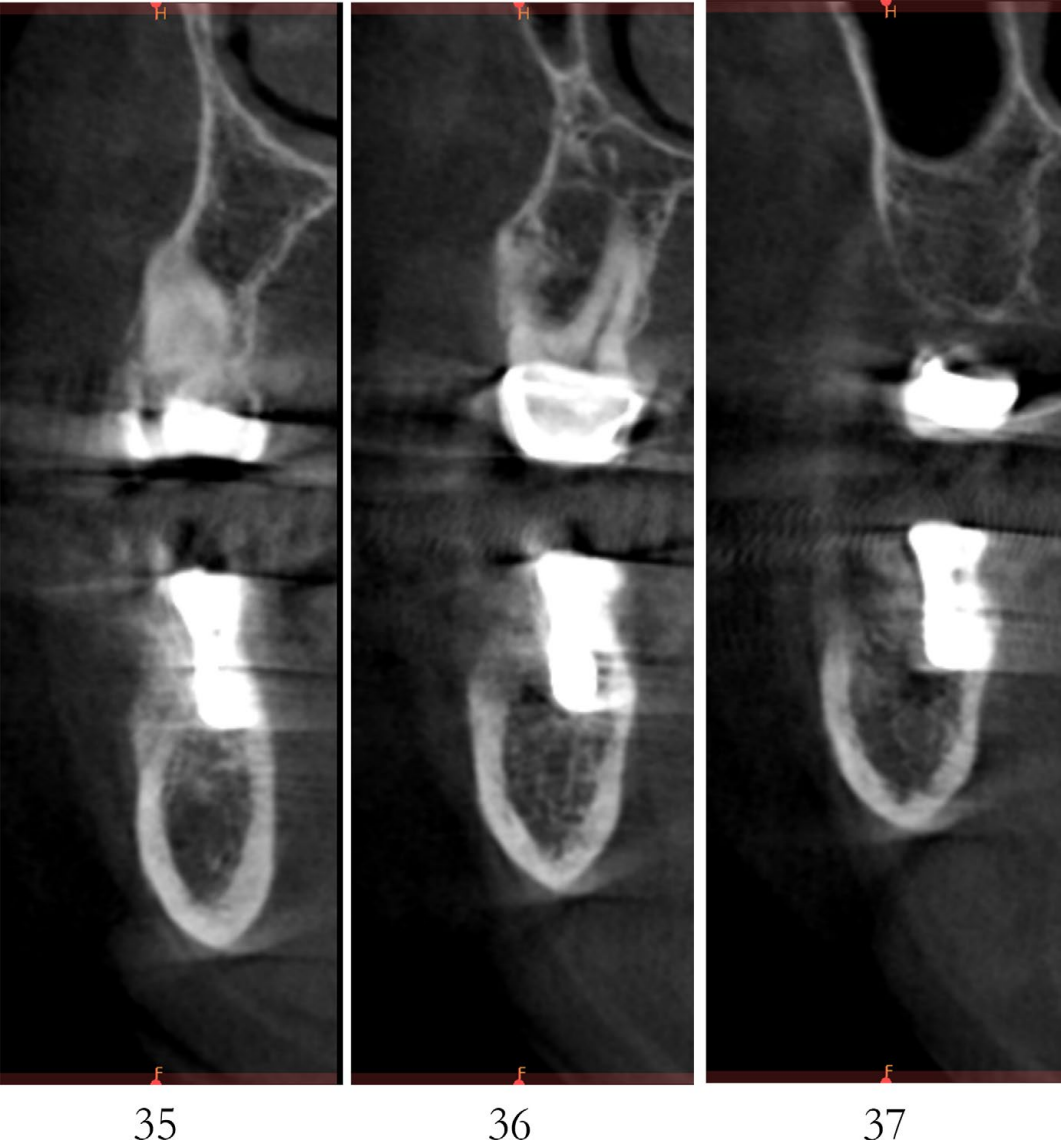
CBCT scanning 6 mo after the bone operation

**Figure 29:**
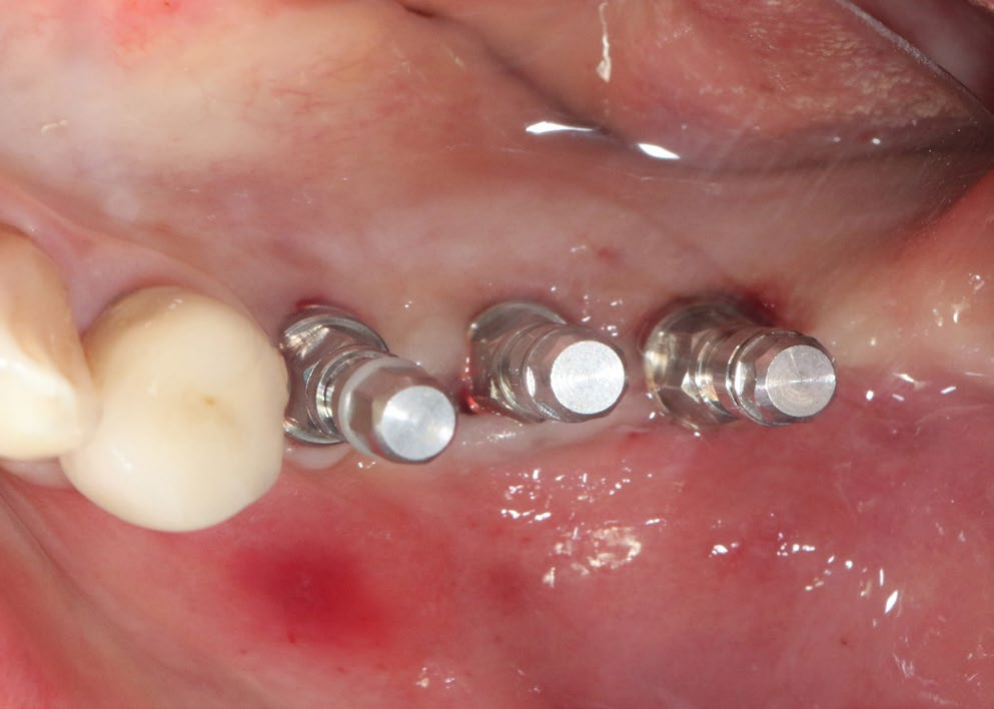
Three Straumann implants installed

**Figure 30:**
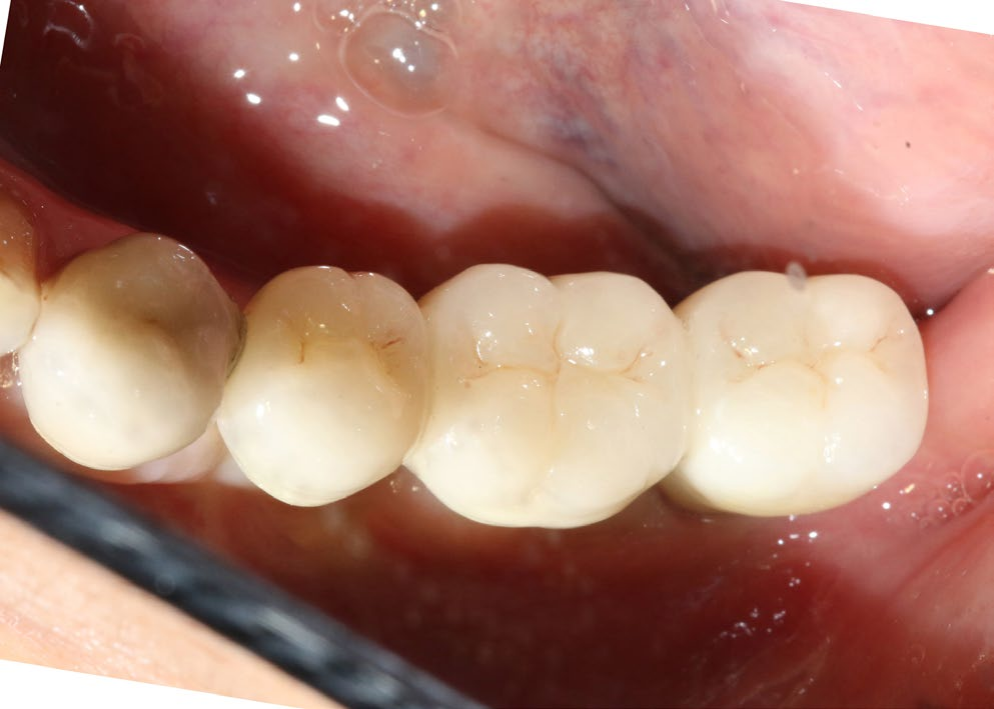
Final restoration

**Figure 31:**
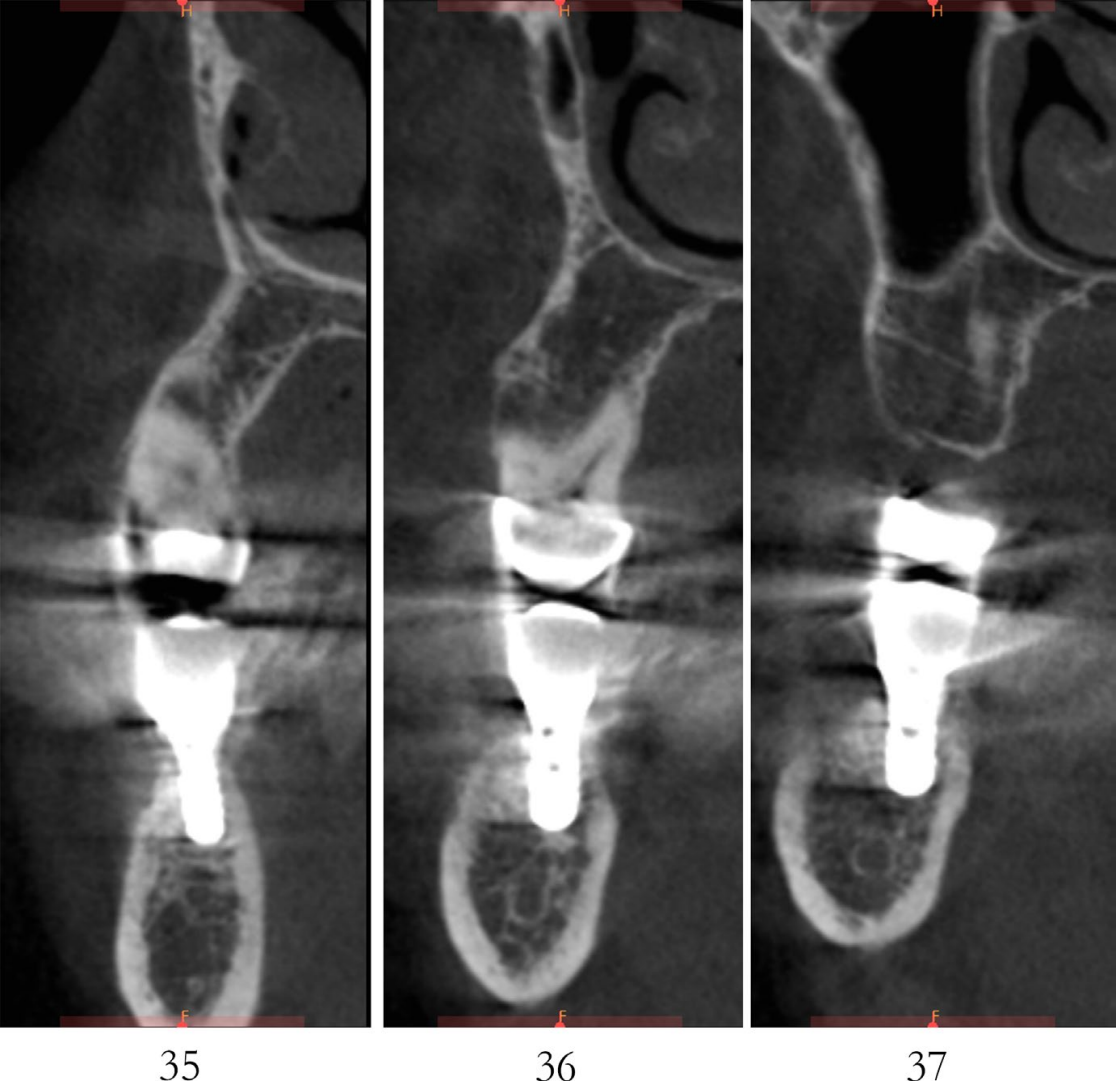
CBCT scanning the day at final restoration

After 6 months, CBCT revealed a horizontal augmentation of 2‐3 mm in the alveolar ridge (Figure 28), and conventional dental implantation (#35:3.3 mm(ф) × 8 mm(L), #36:4.1 mm(ф) × 8 mm(L), #37:4.1 mm(ф) × 8 mm(L); Straumann®, Switzerland) (Figure 29) was performed under local anesthesia. At 3 months after implant placement, all‐ceramic crowns were made to finish the final prosthodontics (Figures 30 and 31).

### 3.3 Therapeutic outcomes

**Figure 32:**
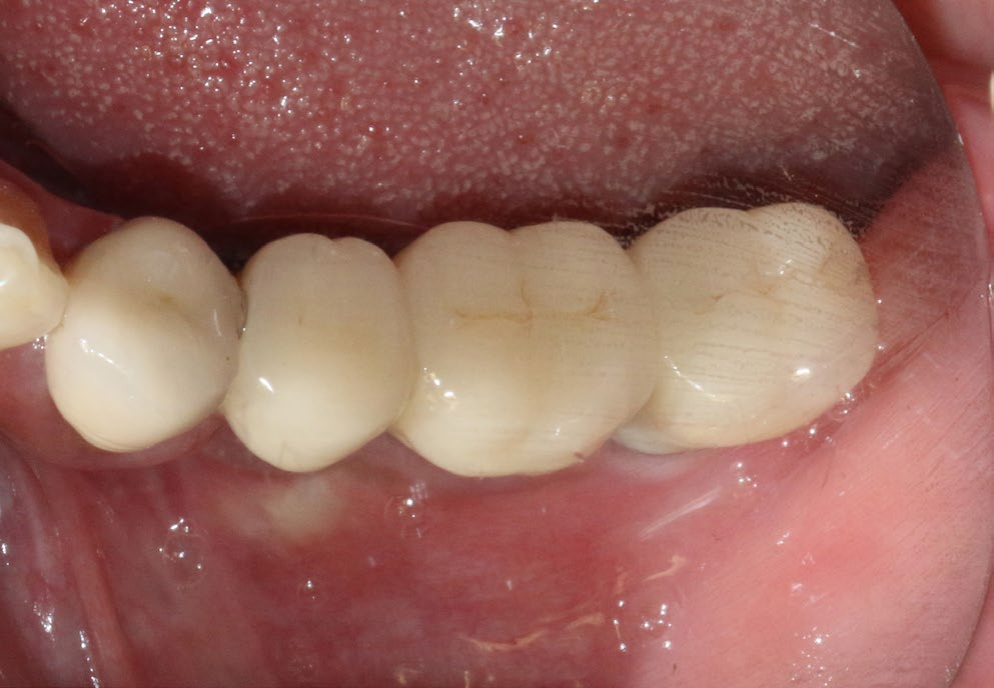
Return visit 2 y after final restoration

**Figure 33:**
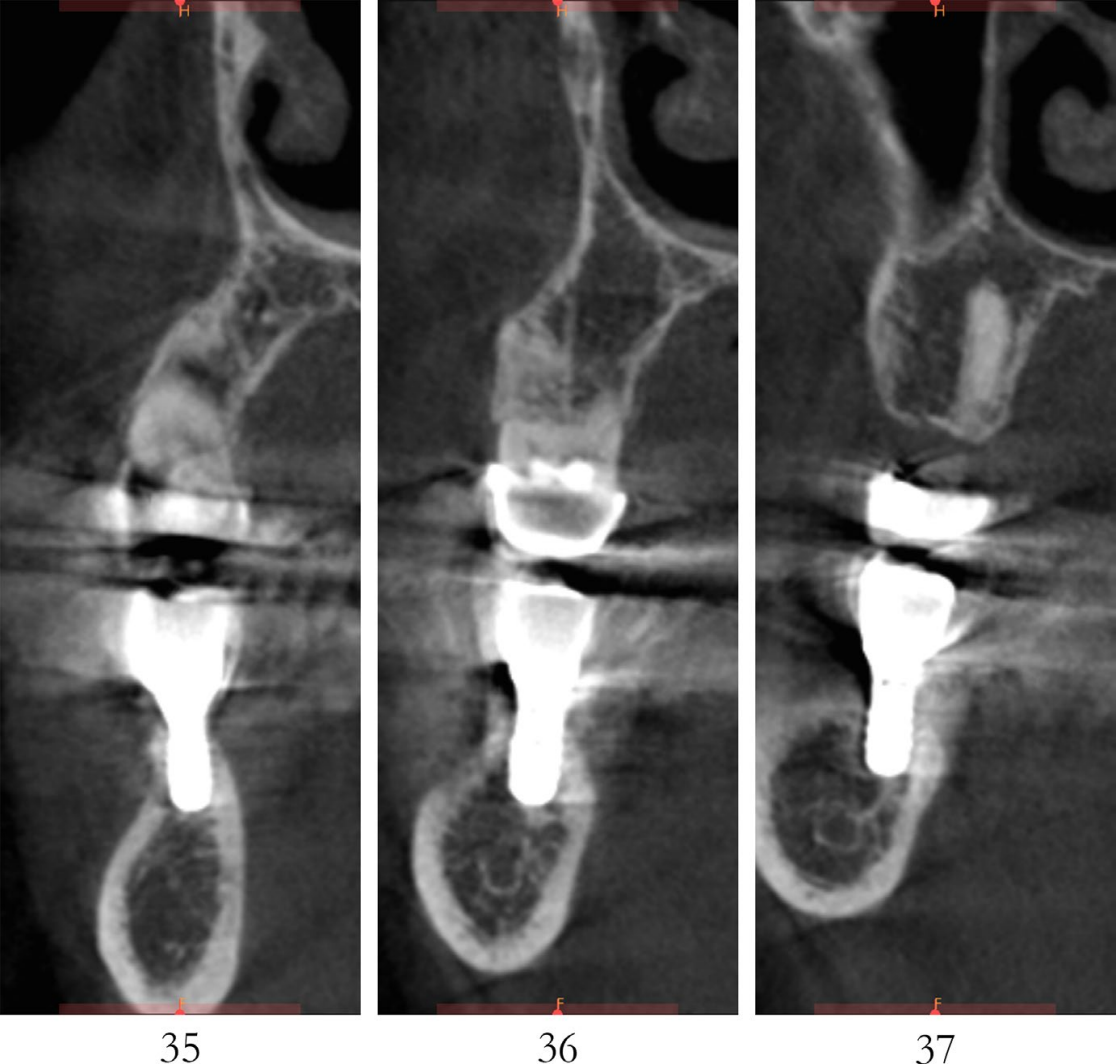
CBCT scaning 2 y after final restoration

A significant bone increment was obtained at the horizontal direction of implant site after the application of modified ultrasonic osteotomy technique. The average bone augmentation at the horizontal direction was approximately 2‐3 mm. The final implant prosthodontics was achieved with a solid and stable bone quantity without obvious bone resorption for 2 years after implant placement (Figures 32 and 33).

## 4 CASE 3

**Figure 34:**
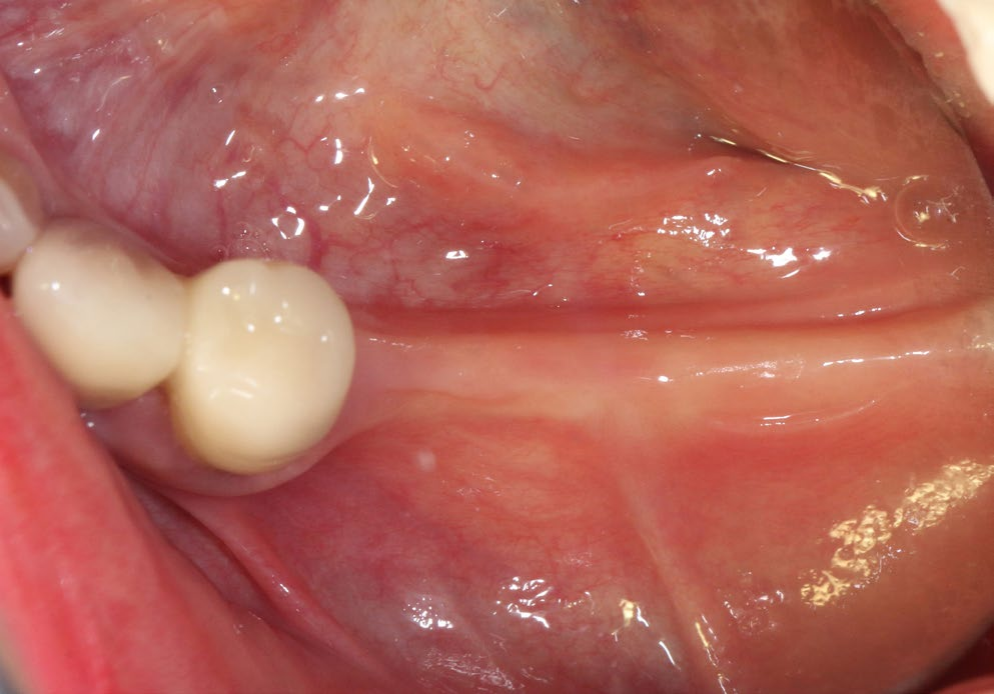
Teeth loss with horizontal bone insufficiency

**Figure 35:**
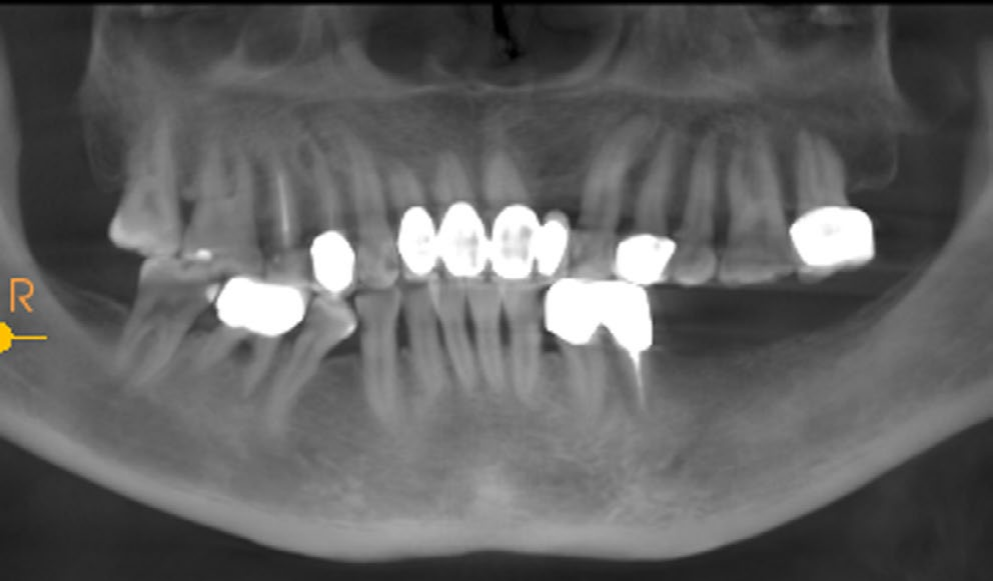
Panoramic view

**Figure 36:**
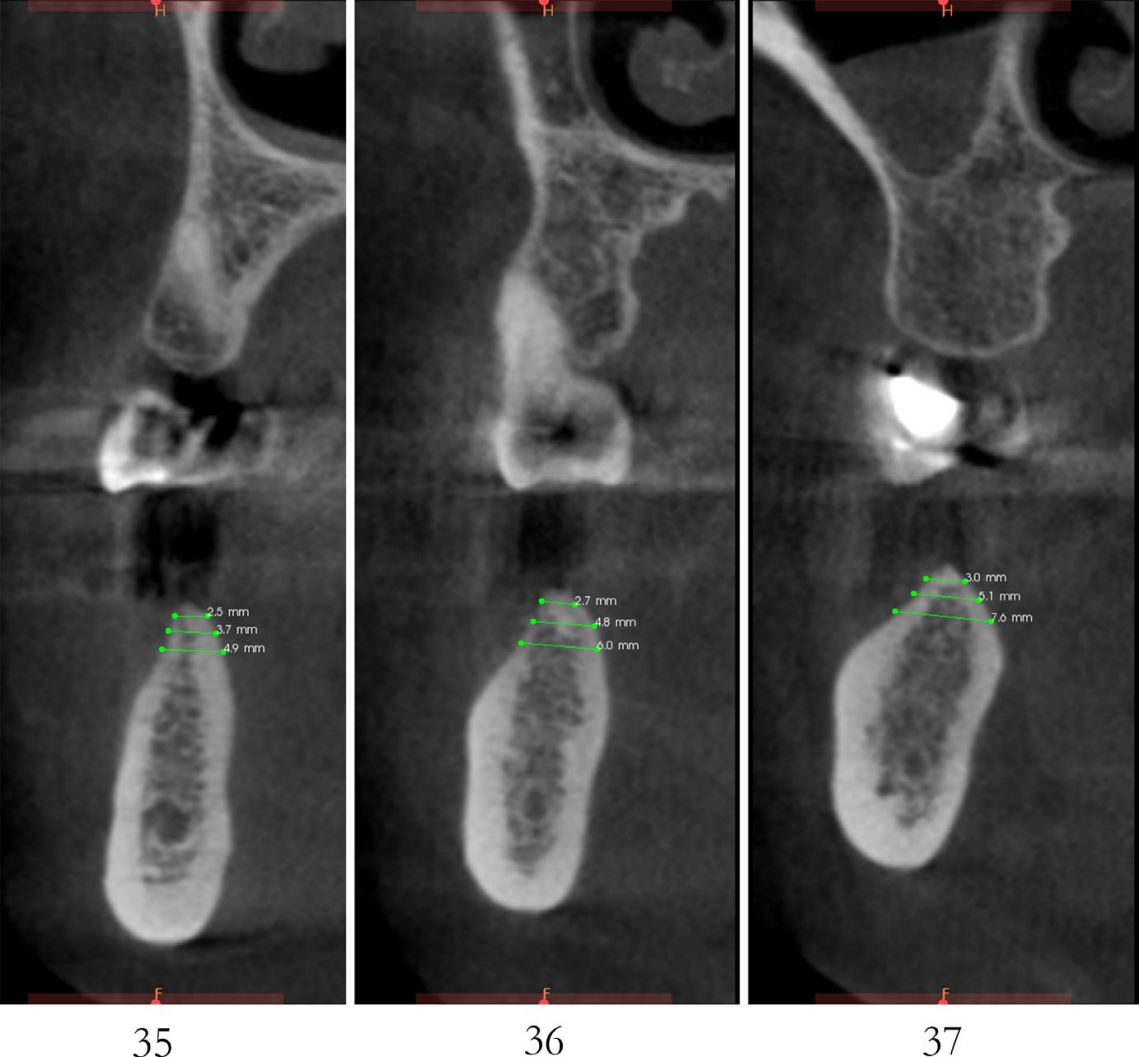
Residual bone crest (#35,#36,#37)

Case 3 was a 42‐year‐old female patient without systemic diseases. With 35, 36, and 37 lost for severe dental caries for over 10 year, the residual alveolar crest for such sites were shown as “blade shaped” (Figures 34, 35, 36).

### 4.1 Treatment plan

Ultrasonic piezosuegery induced bone splitting with simultaneous GBR was designed for this case, and followed by delayed implant placement.

### 4.2 Treatment process

**Figure 37:**
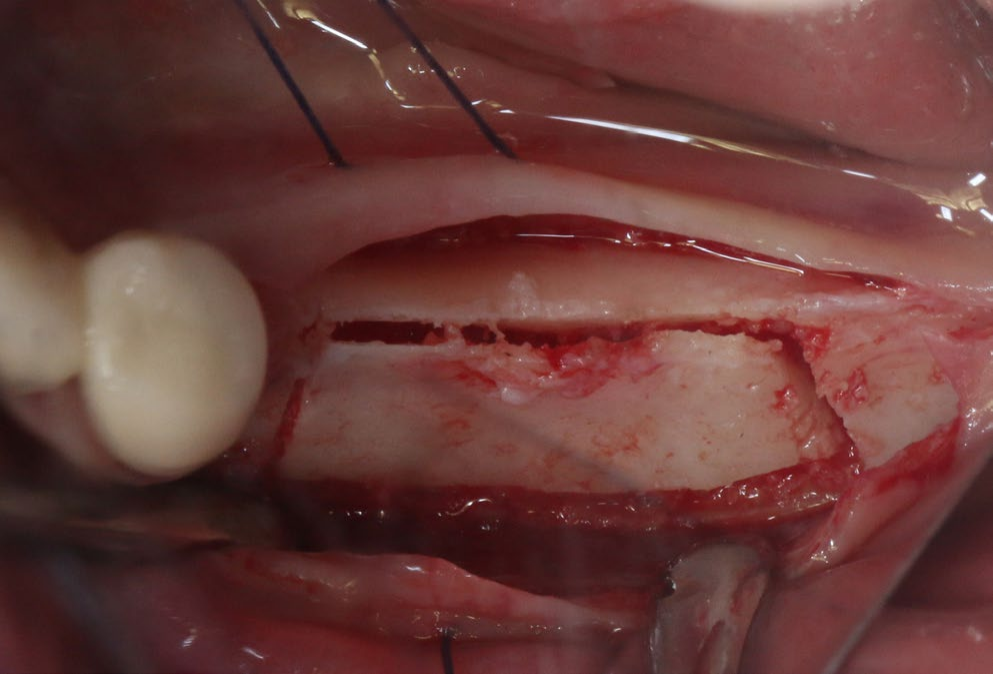
The linear cortical incision by PIEZOSURGERY

**Figure 38:**
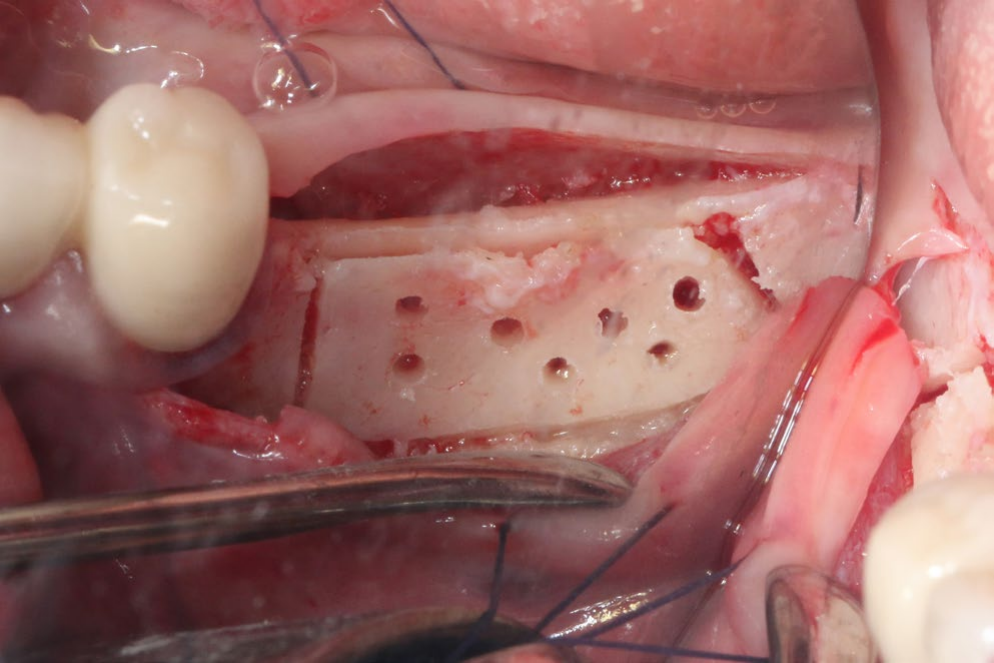
Bleeding holes

**Figure 39:**
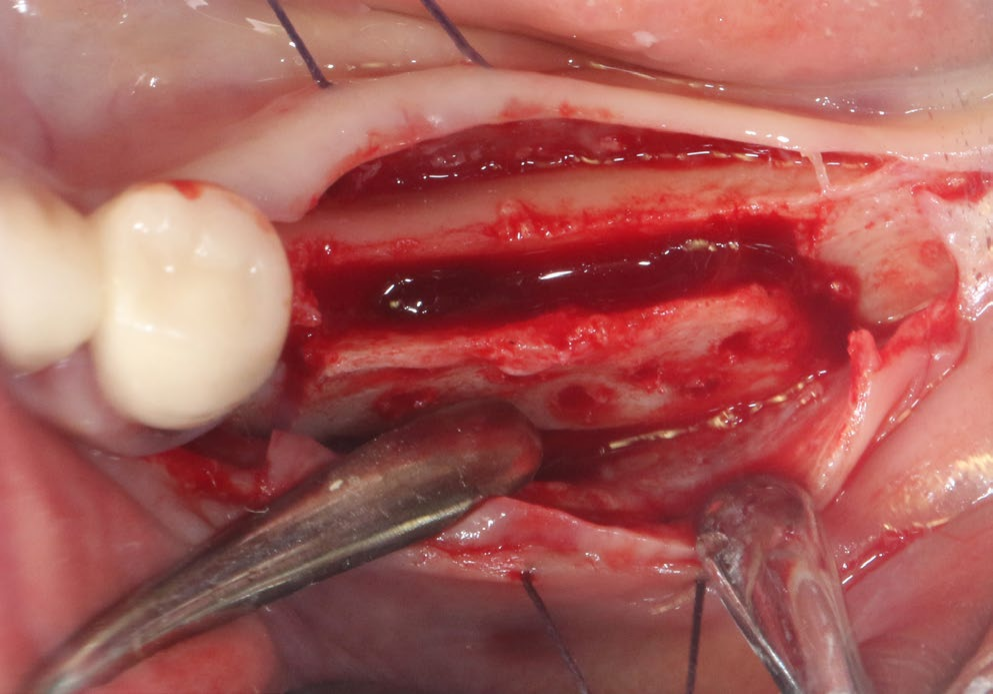
Bone dilatation

**Figure 40:**
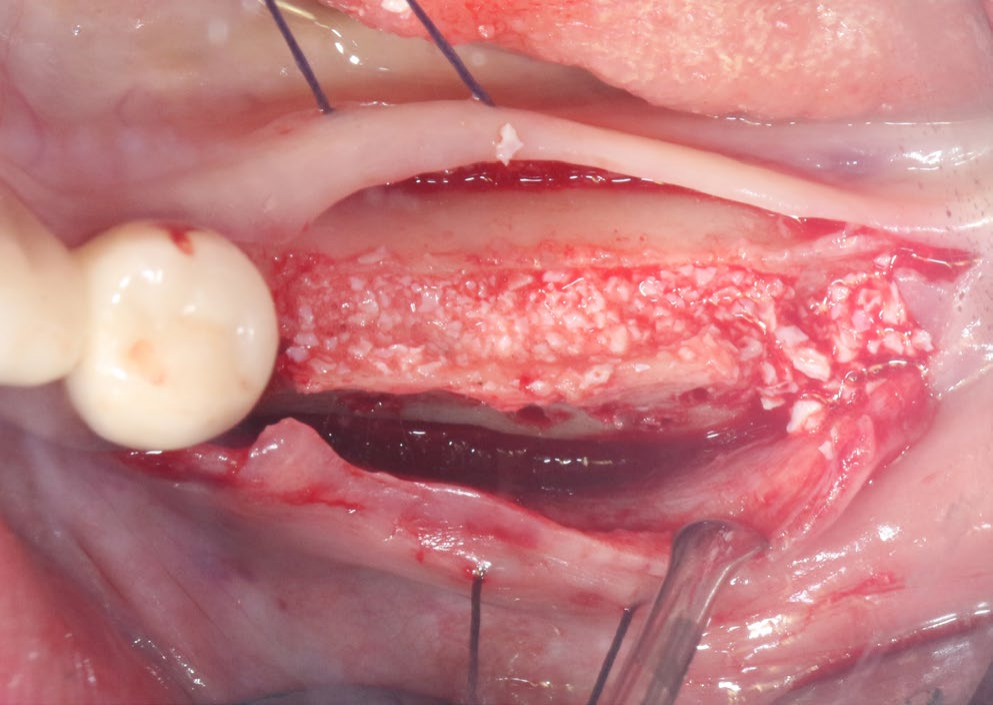
Sandwich bone substitute filling

**Figure 41:**
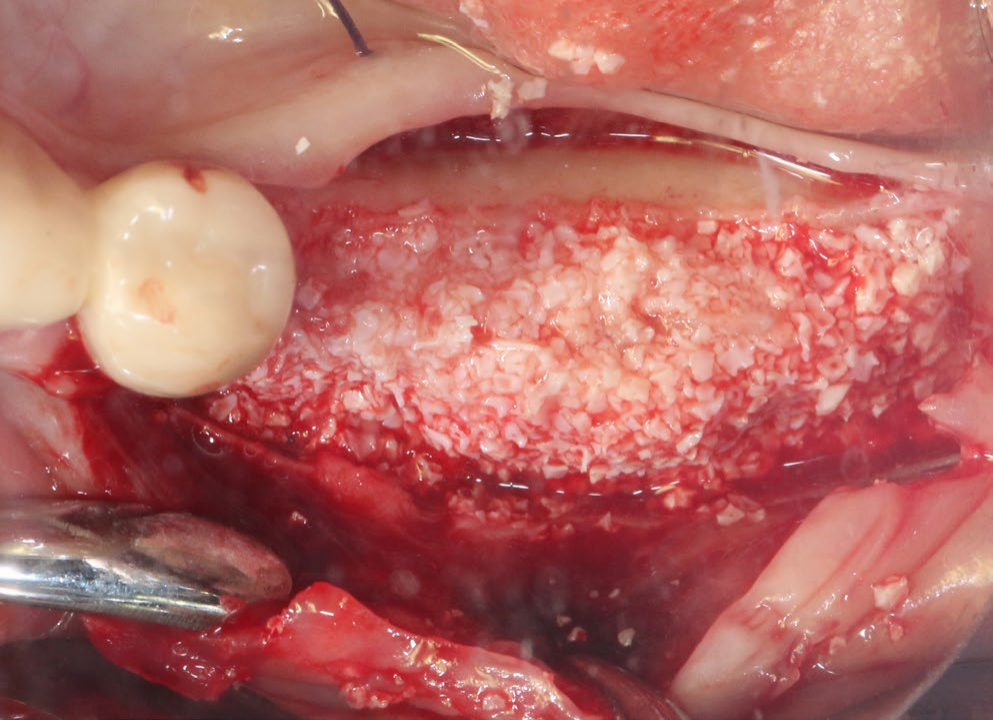
Bone substitute filling buccally

**Figure 42:**
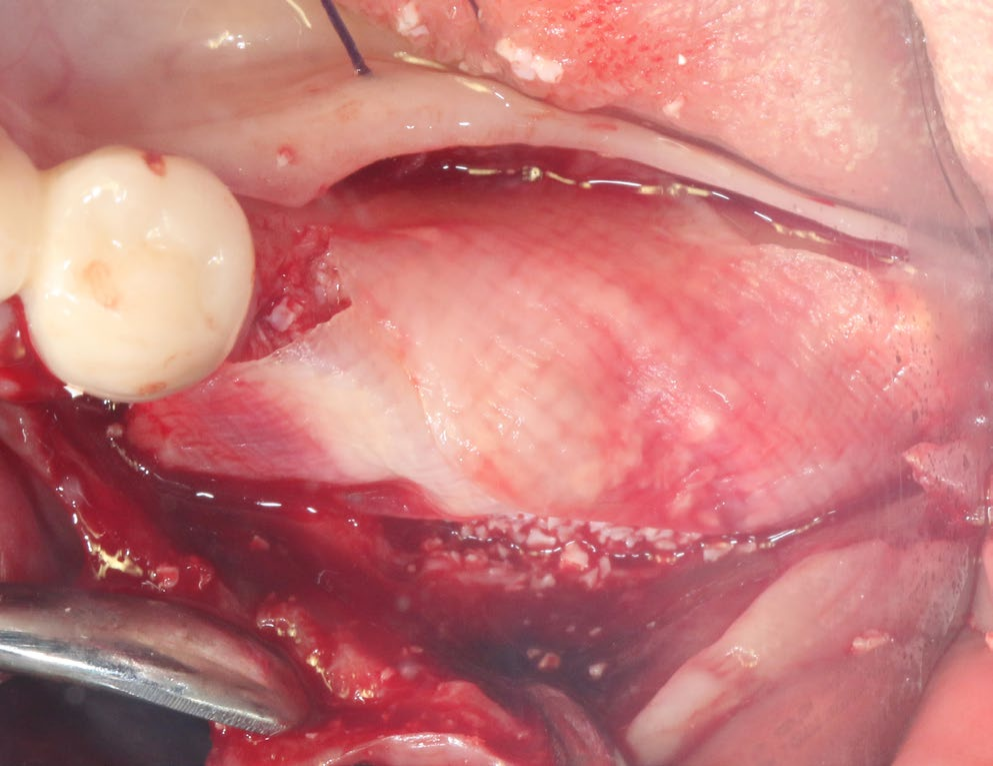
Covered by CGF membrane

**Figure 43:**
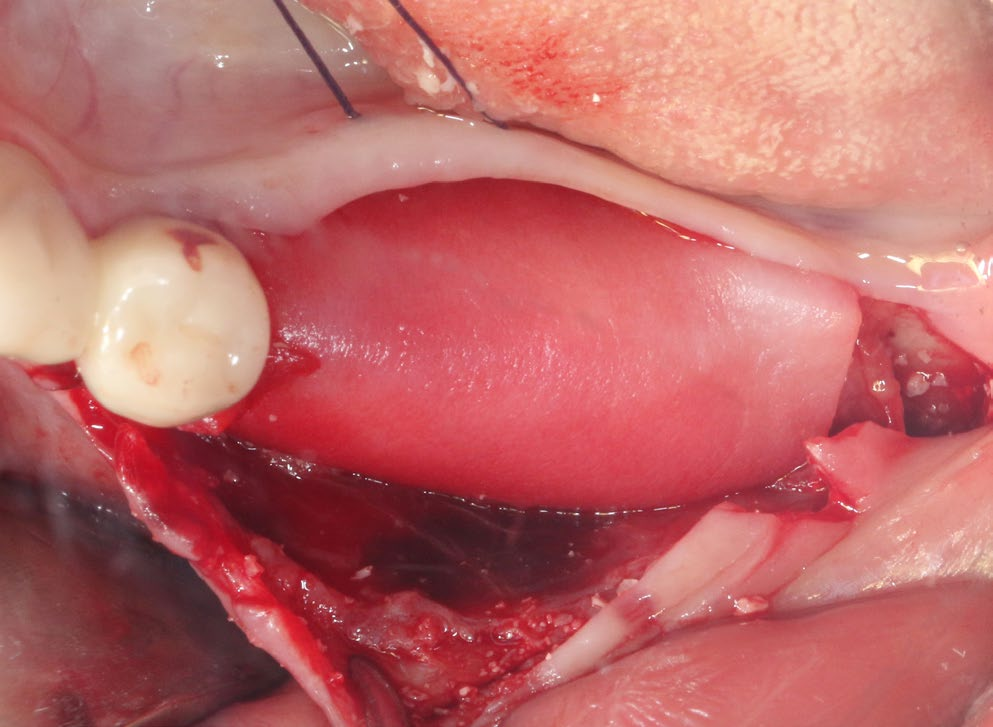
Covered by biofilm

**Figure 44:**
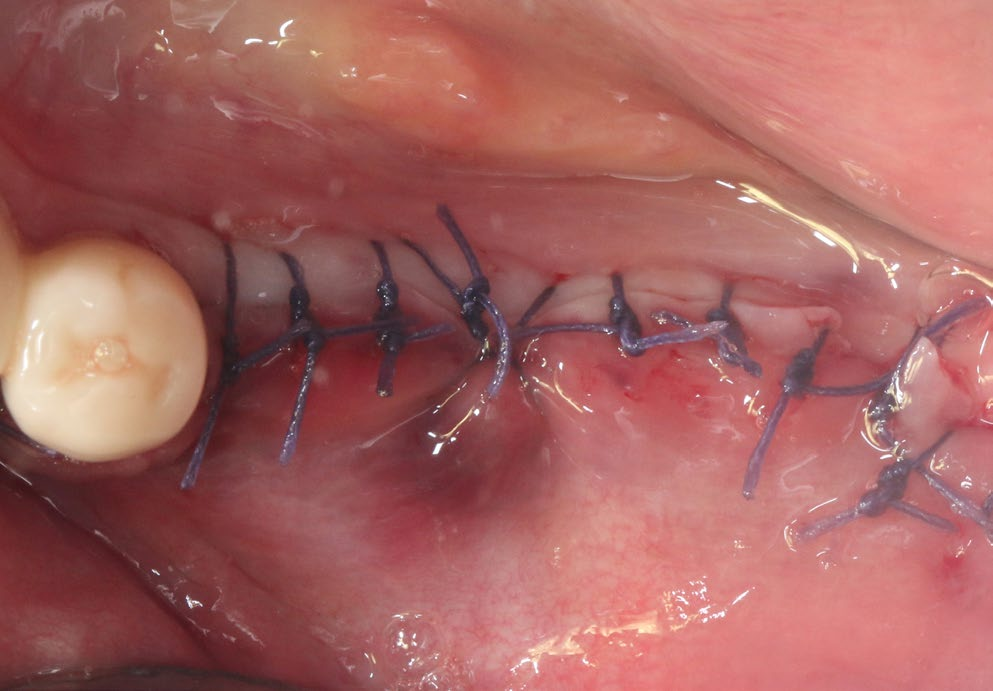
Suture the wound

The same surgical procedure is performed at the site of surgery (35‐37) (Figures 37, 38, 39). After the bone splitting, a Bio‐oss bone substitution was placed into the surgical area (Figures 40 and 41), and two pieces of PRF and biological membrane (Haiao Heal‐ALL, ZH‐BIO) were finally applied to cover the GBR region (Figures 42,43). Other treatments and postoperative instructions were the same as in case 1 and 2 (Figure 44).

**Figure 45:**
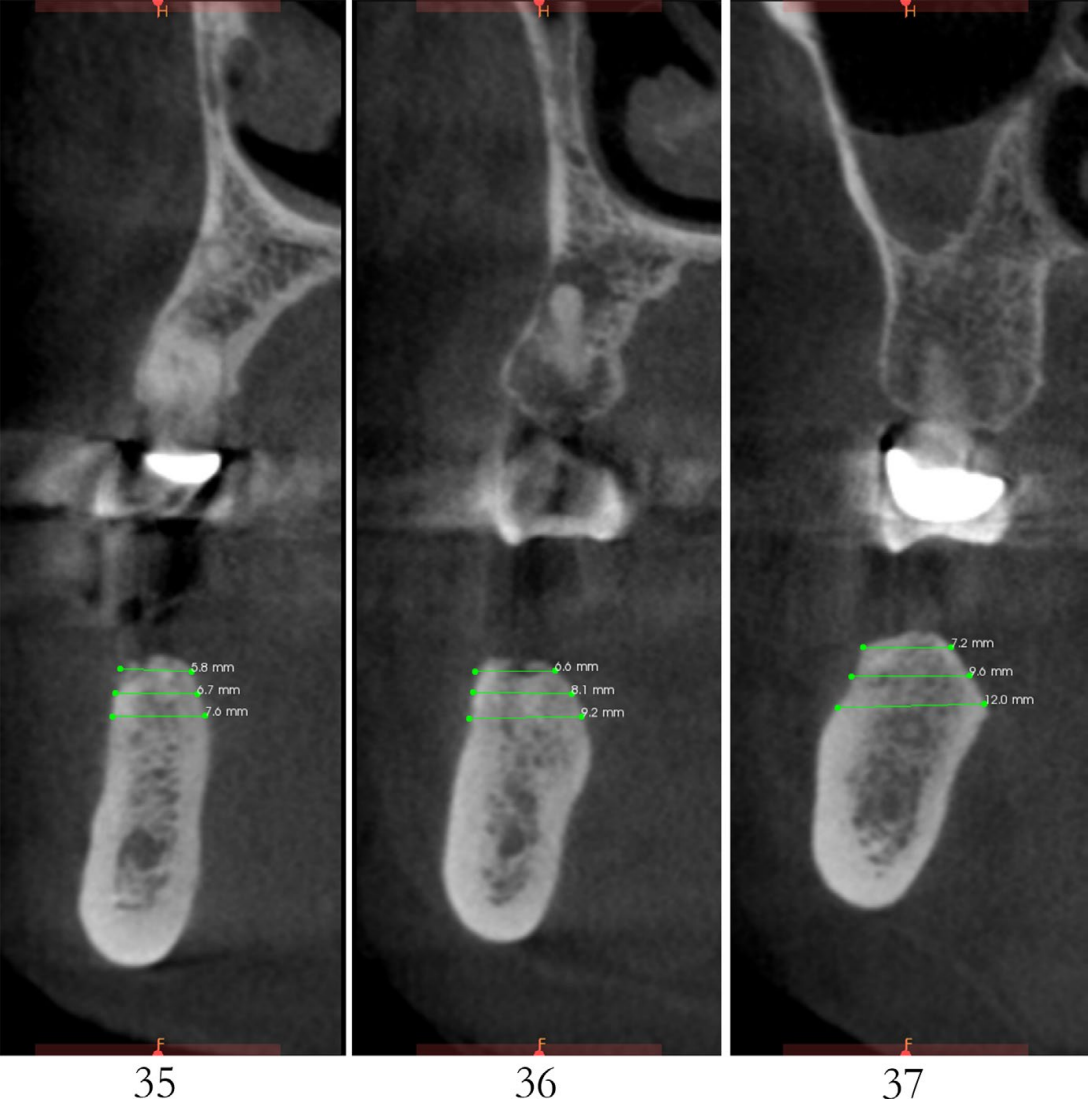
CBCT scanning 6 mo after the bone operation

**Figure 46:**
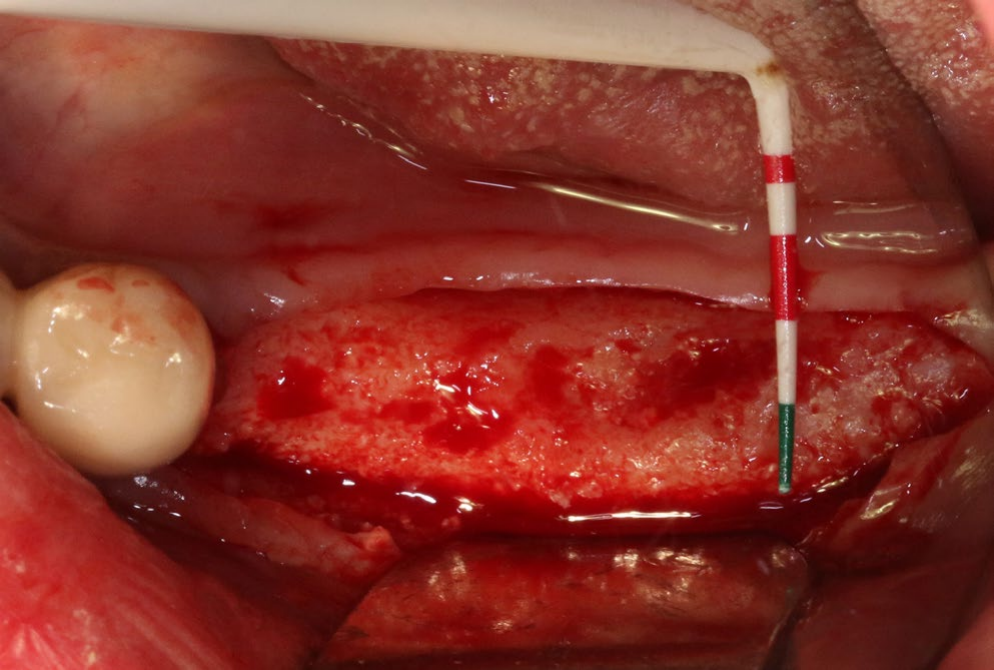
Bone augmentation 6 mo later

**Figure 47:**
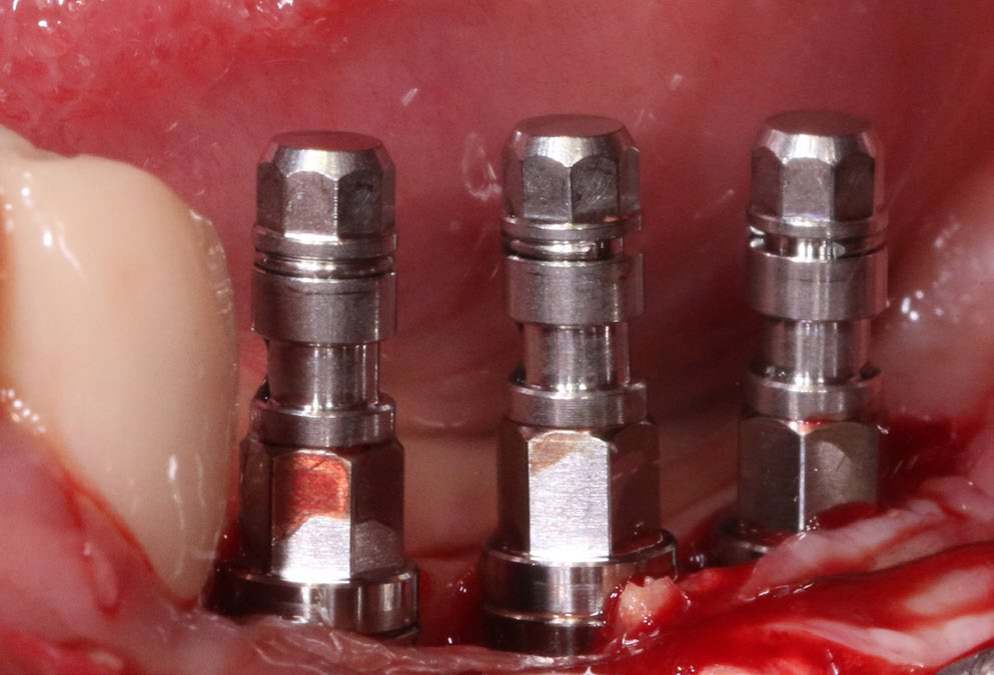
Three Straumann implants installed

**Figure 48:**
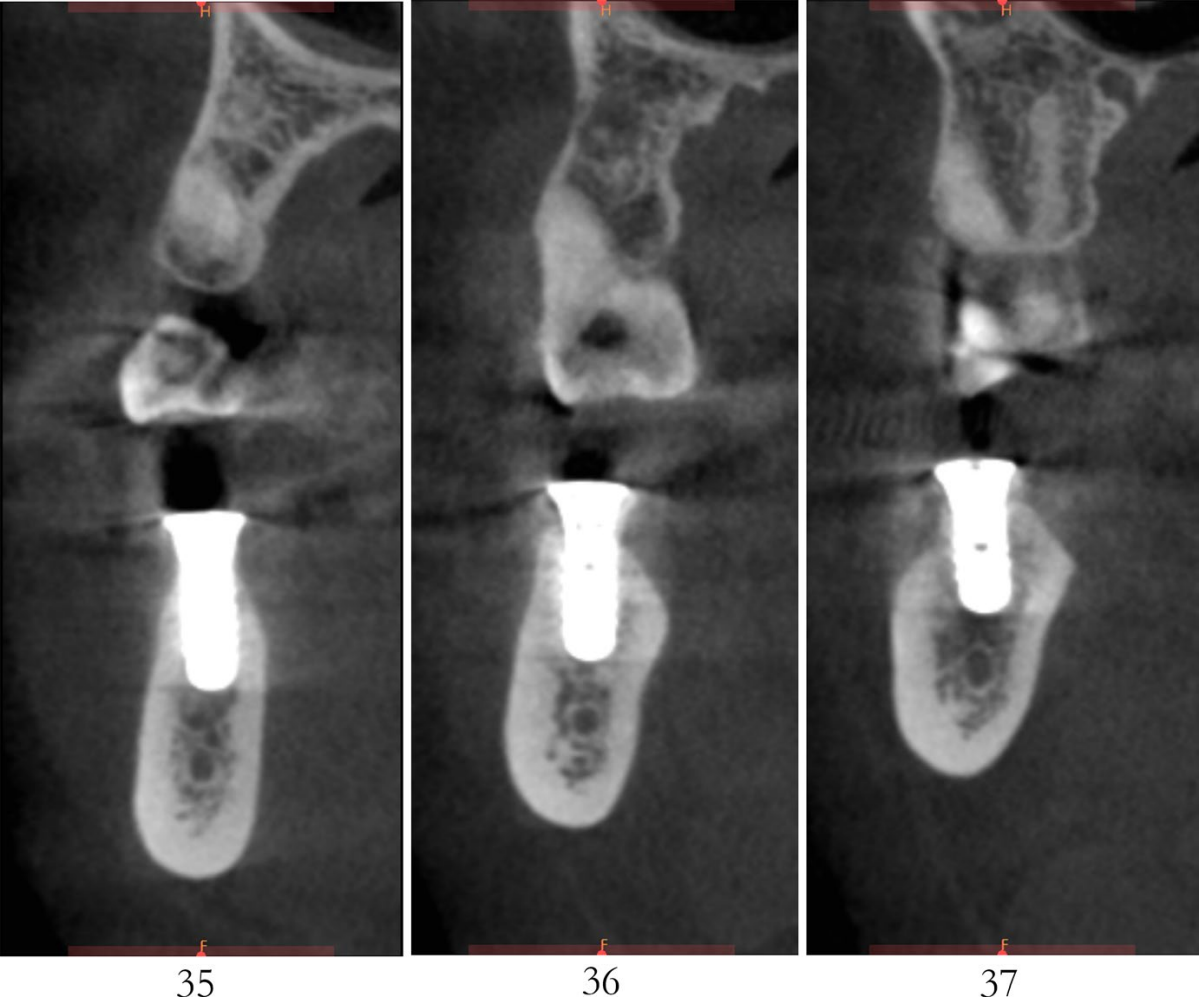
CBCT scanning the day before final restoration

**Figure 49:**
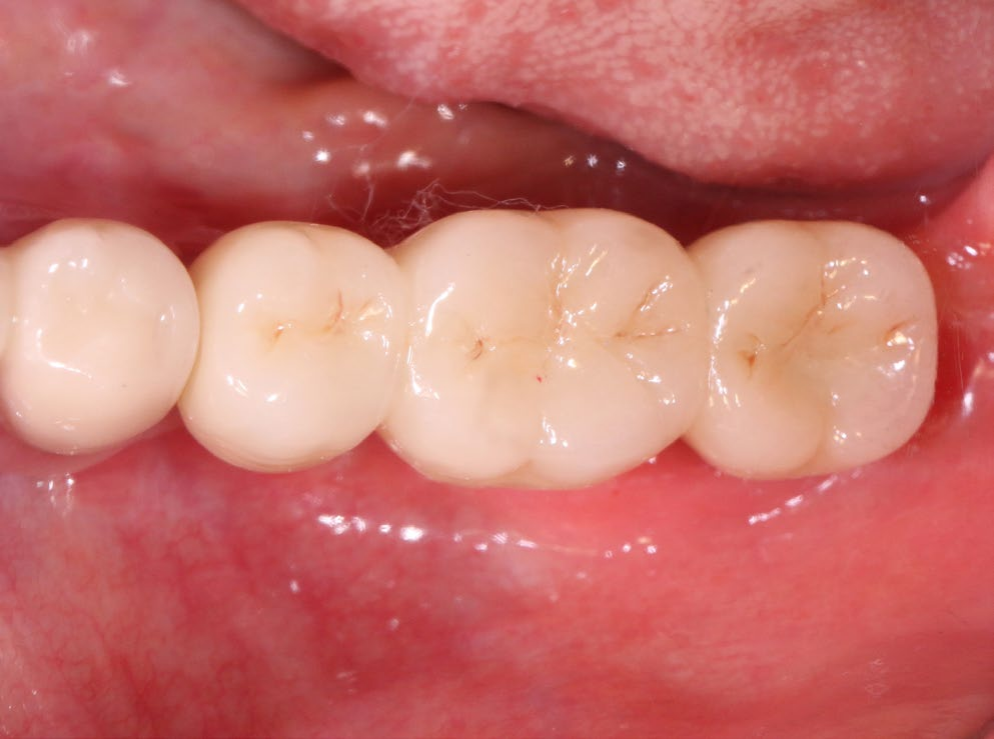
Final restoration

After 6 months, CBCT images indicated an average augmentation of 2‐3 mm in the width of residual alveolar ridge width (Figure 45). Under local anesthesia, a minimally invasive dental implantation technique was performed by nonflap implant placement, three Straumann implants(#35:4.1 mm(ф) × 10 mm(L), #36:4.1 mm(ф) × 10 mm(L), #37:4.1 mm(ф) × 8 mm(L); Straumann^®^) were implanted in the augment alveolar ridge of 35 and 36 (Figures 46 and 47). The X‐ray examination at 3 months after surgery indicated an ideally osseointegration and sufficient bone quantity at the implant‐bone interface (Figures 48 and 49).

### 4.3 Therapeutic effect

**Figure 50:**
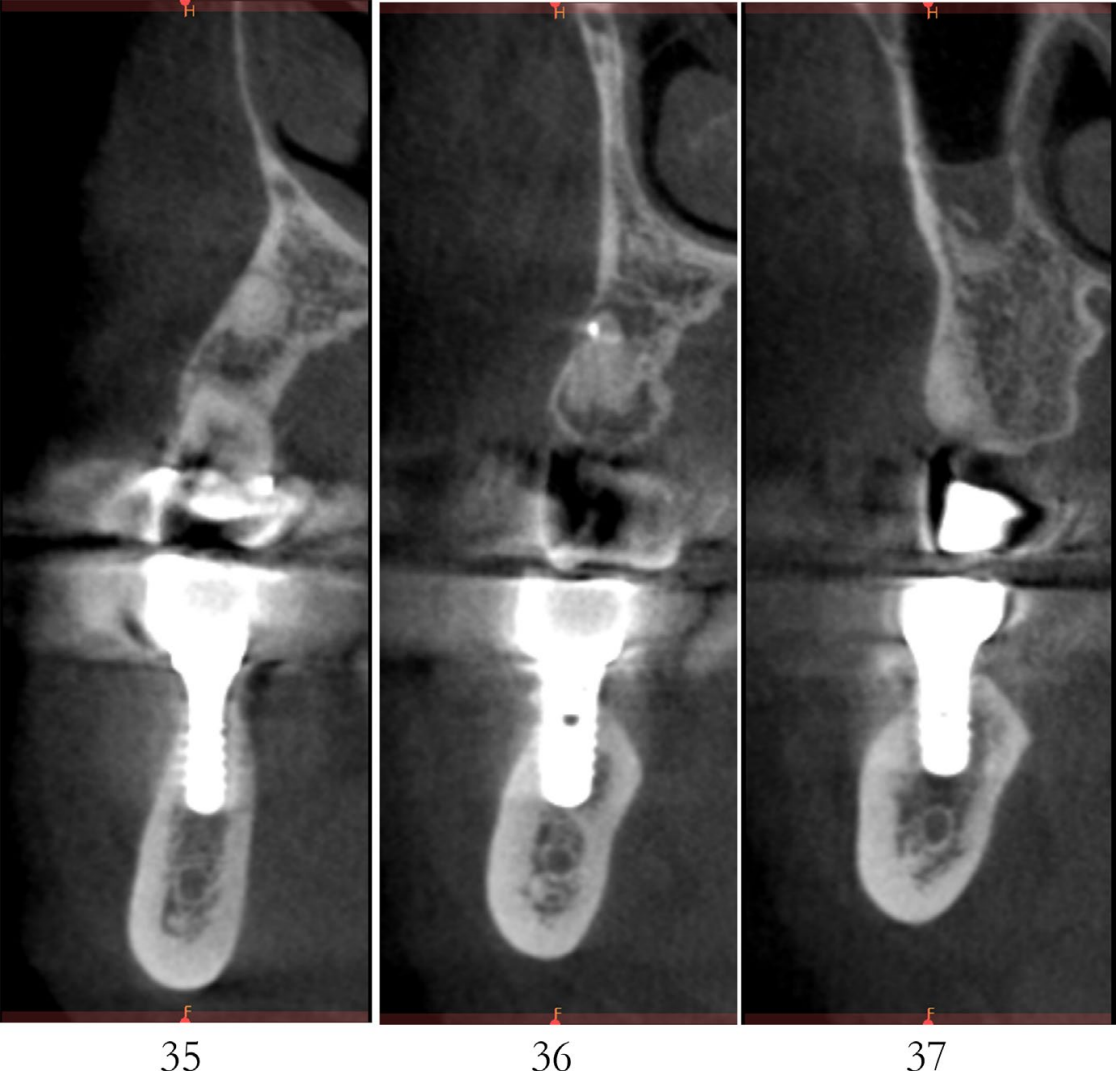
CBCT scanning 2 y after final restoration

**Figure 51:**
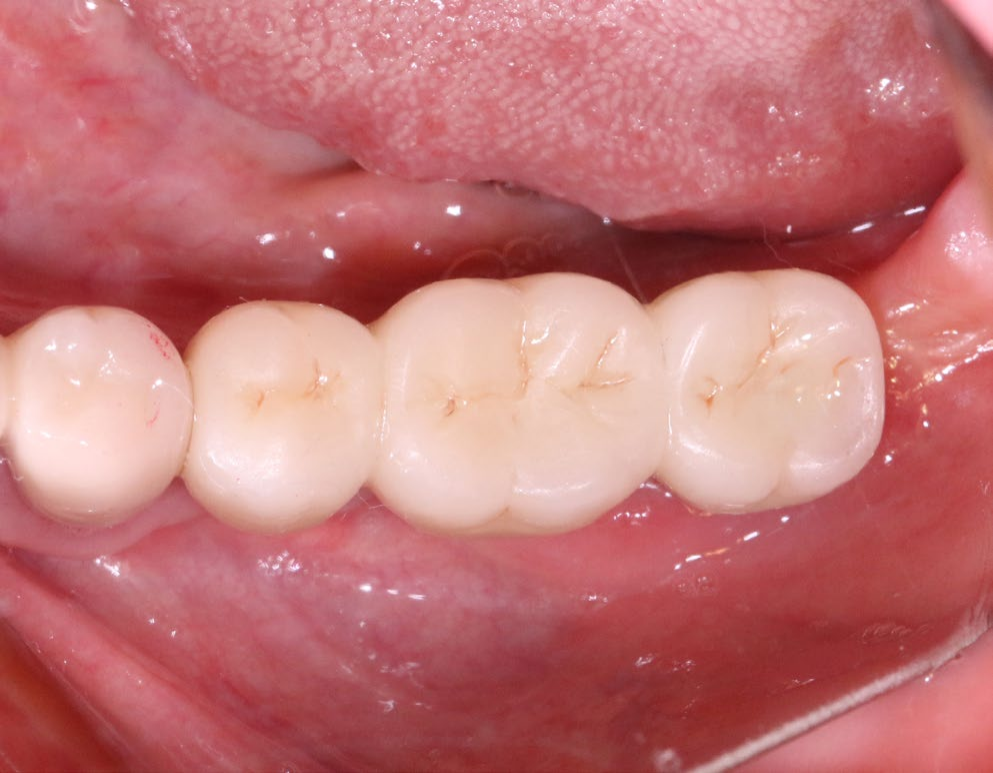
Return visit 2 y after final restoration

The ultrasonic piezosurgery induced bone‐splitting technique dramatically the bone quantity at edentulous region, the horizontal bone quantity was augmented by 2‐3 mm on average (Figures 50 and 51).

## 5 DISCUSSION

To date, various clinical techniques are available for bone regeneration at cases with compromised bone quantity, which include the guided bone regeneration (GBR), onlay grafting, distraction osteogenesis, and bone splitting. GBR is a widely applied and thoroughly investigated technique since its invention,^4^ it has the advantages of lower surgical trauma, and easier to be handled, also is shown with comparatively high success rate; however, the mere application of GBR may result in severe postoperative bone resorption, as Arunjaroensuk^5^ found, this may relate to compromised bone strength and quantity. The onlay bone grafting was shown with a high success rate, and the autologous grafted bone is resistance to future resorption.^6^ However, this technique has severe disadvantages such as aggravated postsurgical trauma, additional surgical area of bone donor sites. As Luca Cordaro et al^7^ reported, when using heterogeneous bone grafting, the infection and other complications are more prone to occur. Distraction osteogenesis also have many advantages such as no need for autologous bone harvesting, a high proportion of new bone in the augmented area, and also makes it possible for the simultaneous soft tissue augmentation which guaranteed an excellent prosthodontic aesthetics.^8^ But the disadvantage is that the size and direction of traction need to be repeatedly adjusted, which means additional times of surgery. In case of compromised bone width, thin implant is an alternative method for implant placement.^9^ However, application of such implants are very limited with many restrictions, in severe bone atrophy cases even the thinnest implant cannot fulfill the criteria that buccal/lingual sides of residual bone should exceeded 1‐1.5 mm.

Bone‐splitting technique creates an affluent alveolar bone width by separating the bone plate into buccal and lingual sides, which can effectively increase the alveolar bone width with lower surgical trauma compared with onlay grafting, and the split buccal/lingual plates could act as frames for maintaining the stability of osteogenic cavity. However, as Simion et al^10^ reported, due to the fact that the cortex of buccal bone plate is thick in the posterior mandible, it is easy to be fractured during bone splitting for the concentration of stress, thus developing a modified bone‐splitting technique with less surgical trauma is necessary. GBR can provide space for osteoblasts by an absorbable membrane which prevents fibroblasts, thus bone tissue can be repaired.^11^ PRF has been widely used in GBR process because of its role in promoting tissue regeneration.^12^ PRF contains a variety of growth factors and cytokines including transforming growth factor‐beta1 (TGF‐β1), platelet‐derived growth factor (PDGF), vascular endothelial growth factor (VEGF), interleukin (IL)‐1β, IL‐4, and IL‐6.^13^ In order to reduce the risk of bone plate fracture, infection and other complications in the process of bone splitting,^14^ and meanwhile, to optimize the process of implantation surgery, the combination of modified bone splitting and GBR techniques can augment the bone width in surgical region safely and effectively.

Mandible is composed of cortex bone and cancellous bone, bone marrow which is rich in blood supply is the majority composition of cancellous bones, moreover, compared with the cortex bones, the cancellous bones are more rapidly in self‐reconstructing and healing. However, the elastic modulus of cancellous bone is shown to be significant lower than that of cortex bone. As a result, the cancellous bone is less likely to be fractured under deforming stress during the bone splitting.

Among the three cases reported in this paper, buccal/lingual sides of residual bone were <1.5 mm (class H‐m according to Wang HVC classification), so an improved alveolar ridge augmentation technique was applied to augment the bone width at mandibular posterior region.^15^ The first stage surgery involved bone splitting and GBR, then the implantion was operated after a 6‐month healing period. After the bone splitting and GBR in the first stage of surgery, the jaw bone was healed and reconstructed to obtain bone augmentation. When the implant was implanted in the second stage, the alveolar bone width could be close to the unabsorbed. So that it could be easier for the second stage. To evenly distribute and induce the splitting stress, mesial and distal incisions were performed perpendicularly to the top incision, and an extra bottom incisions that parallel to the top incision was also applied to induce the splitting stress for safely buccal bone plate expansions. To improve the local blood supply of GBR region, several nourishing holes were drilled to penetrate the buccal bone plate into the cancellous bone region, and the continuity of cancellous bone structure is maintained during the expansion surgery.^16^ These incisions were able to accurately penetrate cortical bone and achieve cancellous bone, by preoperative CBCT measurements and careful intraoperative manipulation. And the scale of ultrasonic bone scalpel provides necessary conditions for accurate cutting. Due to the favorable elastic modulus cancellous bone structures, they are shown to be firm and not easily to be broken during the bone splitting, moreover, to better support the osteogenetic cavity, bone substitutions were also placed between the split buccal‐lingual bone plates, and excessive amount of bone powder was placed on the buccal side to protect the bone plate against postsurgical resorption. After covering by the resorbable membrane and PRF respectively, the wounded region was closed tightly, which can prevent fibroblasts and provide a stable environment for the proliferation and differentiation of osteoblasts. In case 1, the PRF membrane was placed above the absorbable membrane because we were concerned at the beginning of the study that the surgical operation would create tension in the wound and cause poor soft tissue healing. As described above, PRF can promote soft tissue healing, so we tried to obtain soft tissue augmentation by it.^13^ Through postoperative observation, we found that the soft tissue healed well and there was no obvious sign of cracking. So in case 2 we put the absorbable membrane on top of the PRF membrane to get more bone augmentation. To penetrate the cortical bone plates, ultrasonic piezosurgical instruments (PIEZOSURGERY^®^ 3, Mectron S.p.A.) were applied in these surgeries, with are shown with highly accuracy and lower surgical trauma during the bone incisions.^17^ Compared with single‐stage bone splitting which straightforwardly penetrate the cortical and cancellous bones, our methods applied the piezosurgical blade and osteotome to penetrate the cortical and cancellous bones, respectively, this will reduce the risk of surgical complications such as fractures and improve the accuracy during the bone‐splitting surgery. The delayed implantation therapy in these cases provided the mandibles with affluent healing period and thus made it easy to control the position and direction of implant implantation under augmented bone quantity. Compared with an alternative three‐stage surgery therapy, our two‐stage implant surgery can also reduce the number of surgeries, and thus reduces surgical trauma and financial burden for patients.

Combined with the GBR technique, the modified bone‐splitting technique is shown to effectively fill the cleavage gap with sufficient bone substitutions, which provided osteogenic materials for future bone regeneration, and guaranteed a favorable initial stability for the future implant placement.^3^ Through excessive bone substitutions transplanted, the postsurgical resorption will be effectively compensated, the above operations also follow the PASS principle of GBR bone grafting.^18^ Clindamycin, a lincosamide antibiotic, is commonly used to treat gram‐positive aerobic and anaerobic bacterial infections.^19^ Ornidazole is a nitroimidazole which is an antibacterial and antiprotozoal drug used to treat anaerobic enteric protozoa. Also used in the treatment of prophylaxis susceptible anaerobic infections in dental and gastrointestinal surgery.^20^ The risk of infection can be avoided by prophylactic use of antibiotics (clindamycin and ornidazole). Finally, all cases are indicated with ideal bone quantity after a period of healing time, and a robust and stable osseointegration is achieved at bone‐implant interface.

Moreover, for fear of excessive economic burdens and extra surgical trauma by multiple surgeries, none of these 3 cases proceeded soft tissue transplantation and temporary prosthodontics for gingival induction remolding, etc This may limited the final aesthetic outcomes of the implant prosthodontics. In the future cases, we may combine the modified bone‐splitting technique with the soft tissue transplantation to get a sufficient quantity in both soft and hard tissue for ideal implant prosthodontics.

## 6 CONCLUSION

Modified bone splitting can take advantages of the favorable elastic modulus of cancellous bone, and reduce surgical complications such as trauma and fractures. A combination of piezosurgery and GBR techniques can reduce surgical injury and guaranteed an ideal outcome for GBR.

## CONFLICT OF INTEREST

No declared.

## AUTHOR CONTRIBUTIONS

SC, HX, XL and TG: performed the operation and the restoration concerning about these cases and were major contributors in writing the manuscript. TZ: took and collected all the pictures. YZ: designed the work. All authors read and approved the final manuscript and contributed equally to this work.
